# Supplementary material for: Trajectory inference from single-cell genomics data with a process time model
Source: PLoS Comput Biol. 2025 Jan 21;21(1):e1012752. doi: 10.1371/journal.pcbi.1012752 (PMC11760028; doi:10.1371/journal.pcbi.1012752)
Supplement: S1 Text — (PDF) [file pcbi.1012752.s001.pdf]

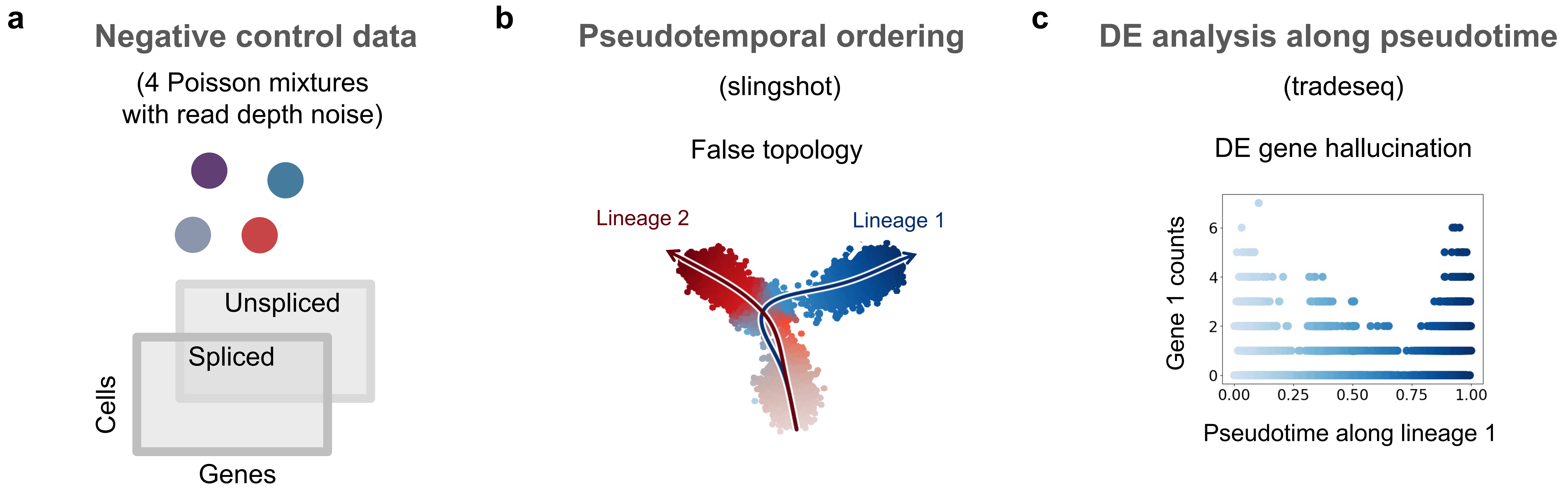
a
Negative control data
(4 Poisson mixtures
with read depth noise)
Unspliced
Spliced
Cells
Genes
b
Pseudotemporal ordering
(slingshot)
False topology
Lineage 2
Lineage 1
c
DE analysis along pseudotime
(tradeseq)
DE gene hallucination
Gene 1 counts
0
2
4
6
0.00
0.25
0.50
0.75
1.00
Pseudotime along lineage 1
