# Supplementary material for: Trajectory inference from single-cell genomics data with a process time model
Source: PLoS Comput Biol. 2025 Jan 21;21(1):e1012752. doi: 10.1371/journal.pcbi.1012752 (PMC11760028; doi:10.1371/journal.pcbi.1012752)
Supplement: S2 Fig — a) The two trajectory structures used in simulations. b) Estimation errors of different parameter sets. For time, error is root mean square error. For α , β , γ, error is mean normalized error as described in the Section Simulations. c) Absolute errors with respect to the true values of parameters. (PDF) [file pcbi.1012752.s003.pdf]

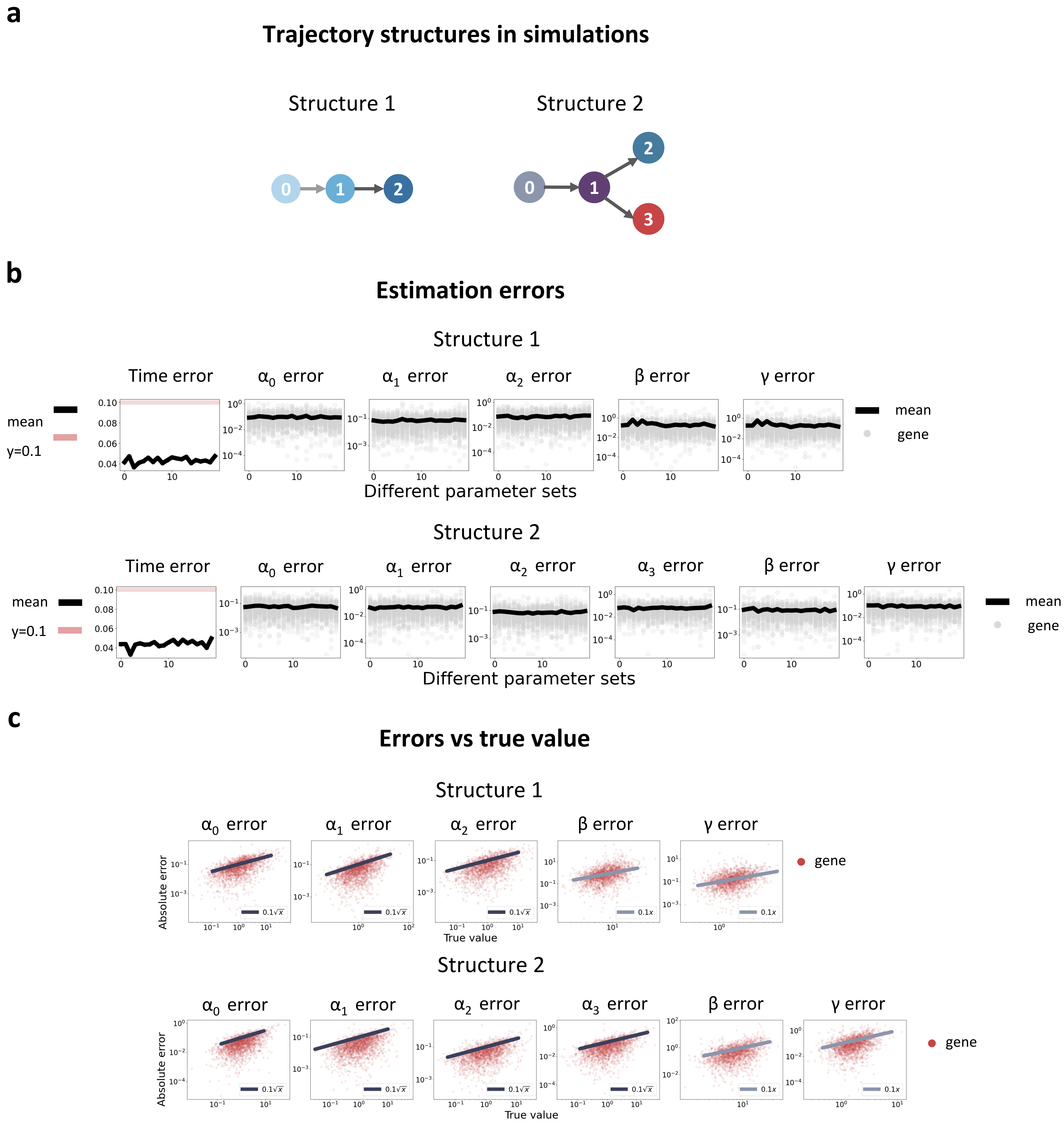
a
Trajectory structures in simulations
Structure 1
Structure 2
b
Estimation errors
Structure 1
Time error
α0 error
α1 error
α2 error
β error
γ error
mean
y=0.1
gene
Different parameter sets
Structure 2
α3 error
c
Errors vs true value
Absolute error
True value
0.1√x
0.1x
