# Supplementary figures and images for: Trajectory inference from single-cell genomics data with a process time model
Source: PLoS Comput Biol. 2025 Jan 21;21(1):e1012752. doi: 10.1371/journal.pcbi.1012752 (PMC11760028; doi:10.1371/journal.pcbi.1012752)

a

## Estimation errors

### Structure 1

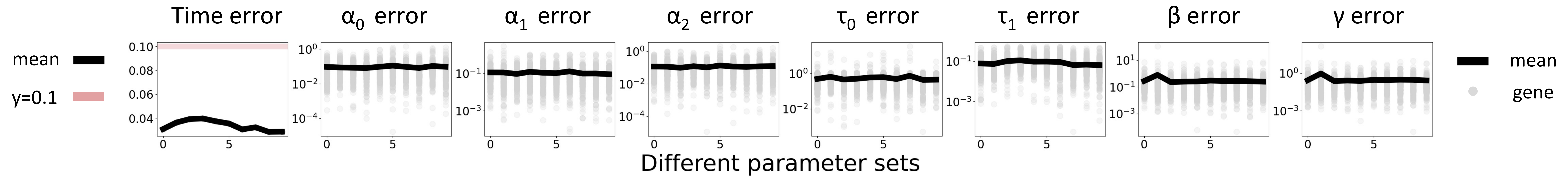

### Structure 2

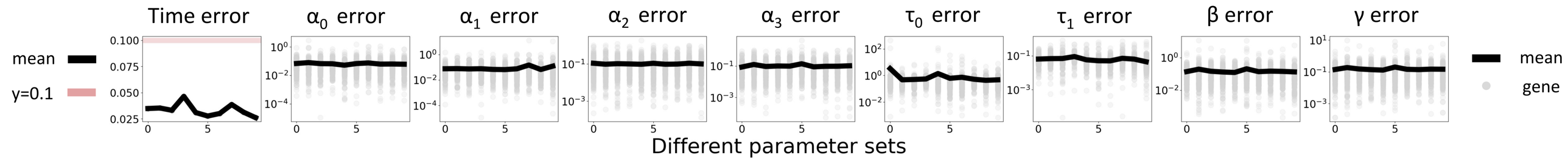

b

## Errors vs true value

### Structure 1

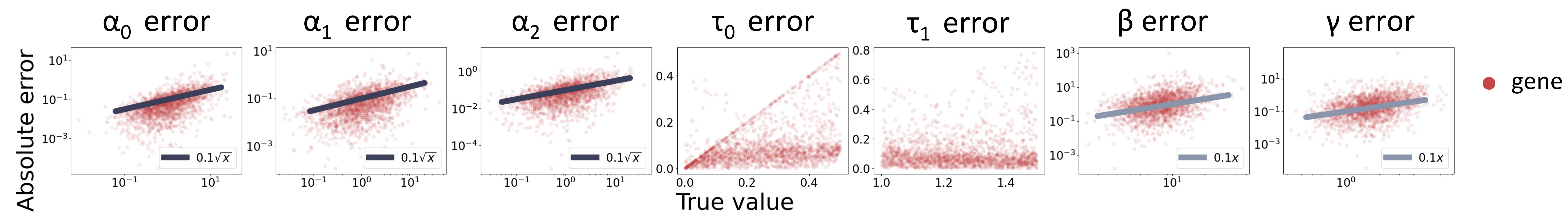

### Structure 2

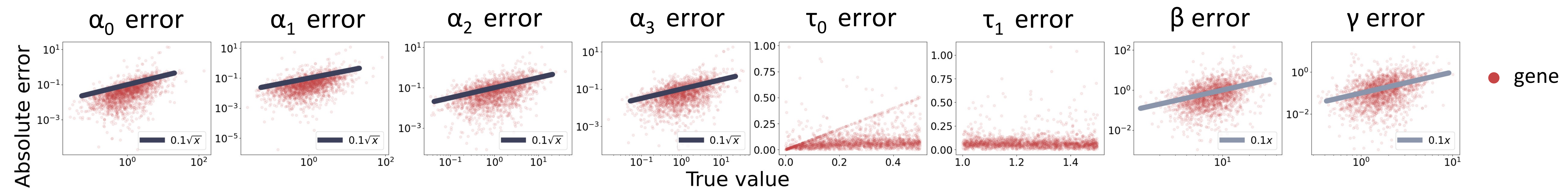

Supplement: S3 Fig — Estimation errors of the desynchronized model tested on simulations with trajectory structures in S2a Fig. a) Errors of different parameter sets. For time, error is root mean square error. For α , β , γ, error is mean normalized error as described in the Section Simulations. b) Absolute errors with respect to the true values of parameters. (PDF) [file pcbi.1012752.s004.pdf]

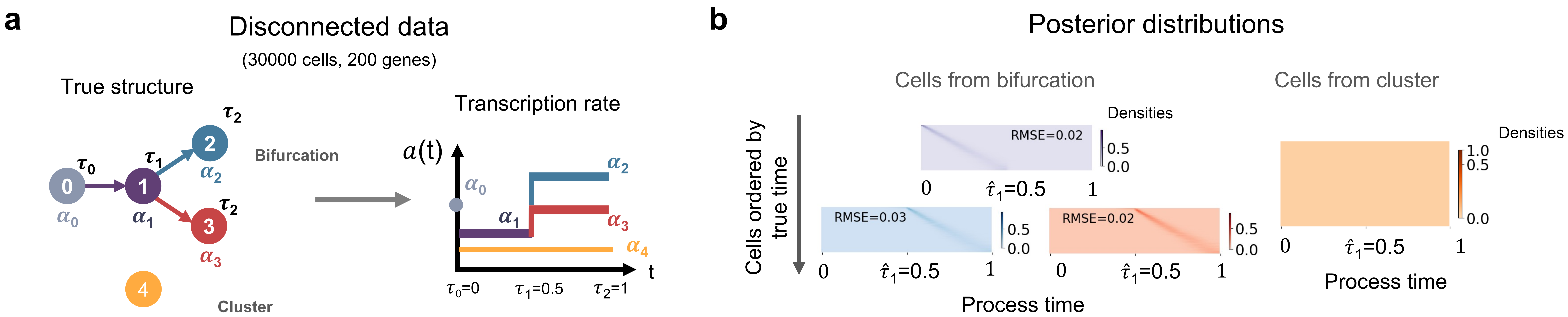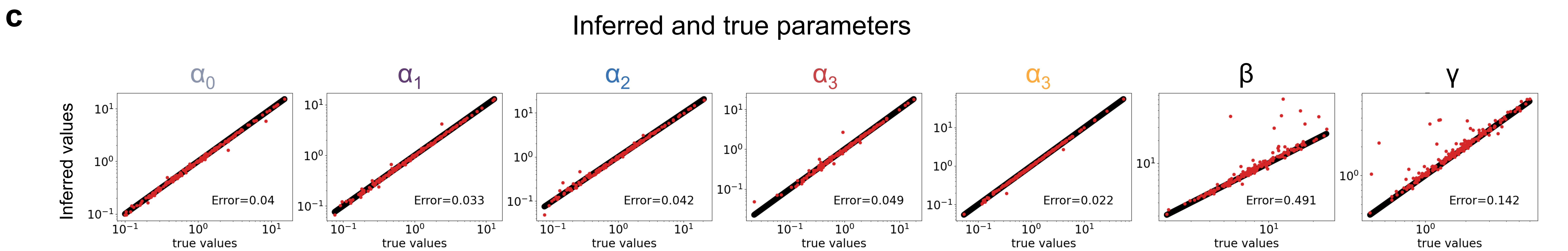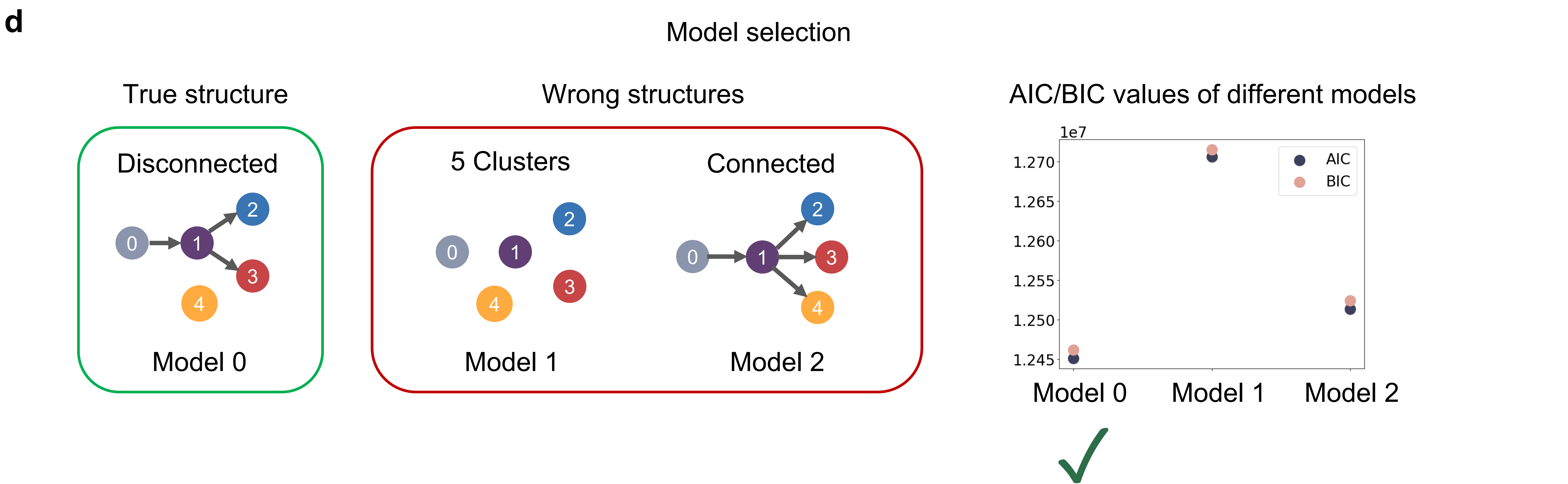

Supplement: S5 Fig — a The ground truth trajectory structure. A subset of cells is from a bifurcation trajectory and the other cells are from a disjoint cluster. For the bifurcation trajectory, cells start from the state 0 and jump to the state 1 at τ0 = 0, and then bifurcate into two lineages with different ending states (2 and 3) at τ1 = 0 . 5. The process ends at τ2 = 1. b Heatmaps of the inferred posterior distributions for cells from both the bifurcation and the cluster. x axis is time grids, and y axis is cells aligned by their true times and grouped by their true lineages. The intensity of color indicates the weights of posterior distributions of cells on the grids. Heatmap of cells from τ0 to τ1 use a purple color palette. Heatmap of cells from τ1 to τ2 of first lineage use blue, and those of second lineage use red. Heatmap of cells from cluster use an orange color palette. RMSE stands for root mean square errors. c Inferred parameters values compared to true values. Error is mean normalized error across genes as described in the Section Simulations. d AIC and BIC of the true model and two wrong models. (PDF) [file pcbi.1012752.s006.pdf]

# Impact of gene numbers on inference accuracy

a

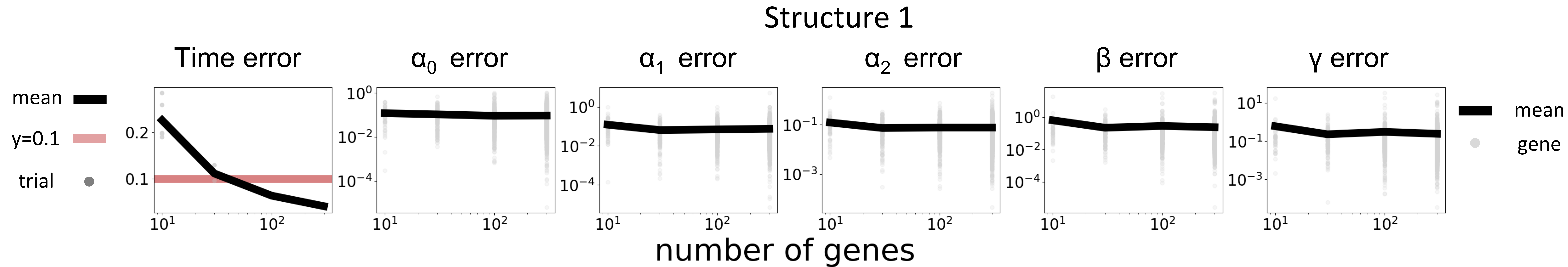

b

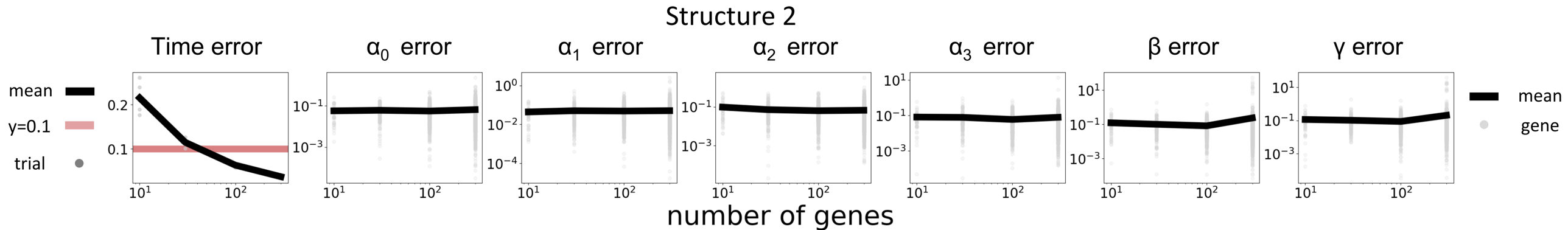

Supplement: S8 Fig — The trajectory structures are the same as in S2a Fig. For time, error is root mean square error. For α , β , γ, error is mean normalized error as described in the Section Simulations. a) Results for trajectory structure 1. b) Results for trajectory structure 2. (PDF) [file pcbi.1012752.s009.pdf]

# Impact of mean counts on inference accuracy

a

Structure 1

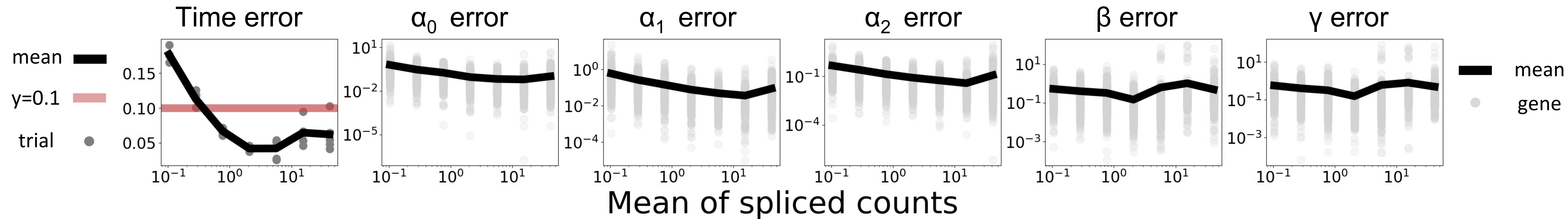

b

Structure 2

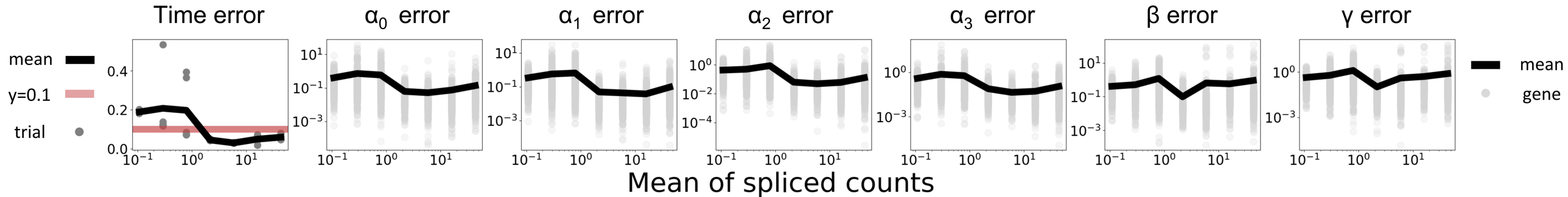

Supplement: S9 Fig — Simulations of different counts mean are generated by scaling the transcription rates while keeping other parameters the same. The trajectory structures are the same as in S2a Fig. For time, error is root mean square error. For α , β , γ, error is mean normalized error as described in the Section Simulations. a) Results for trajectory structure 1. b) Results for trajectory structure 2. (PDF) [file pcbi.1012752.s010.pdf]

**a** Exponential functions with different rate constants

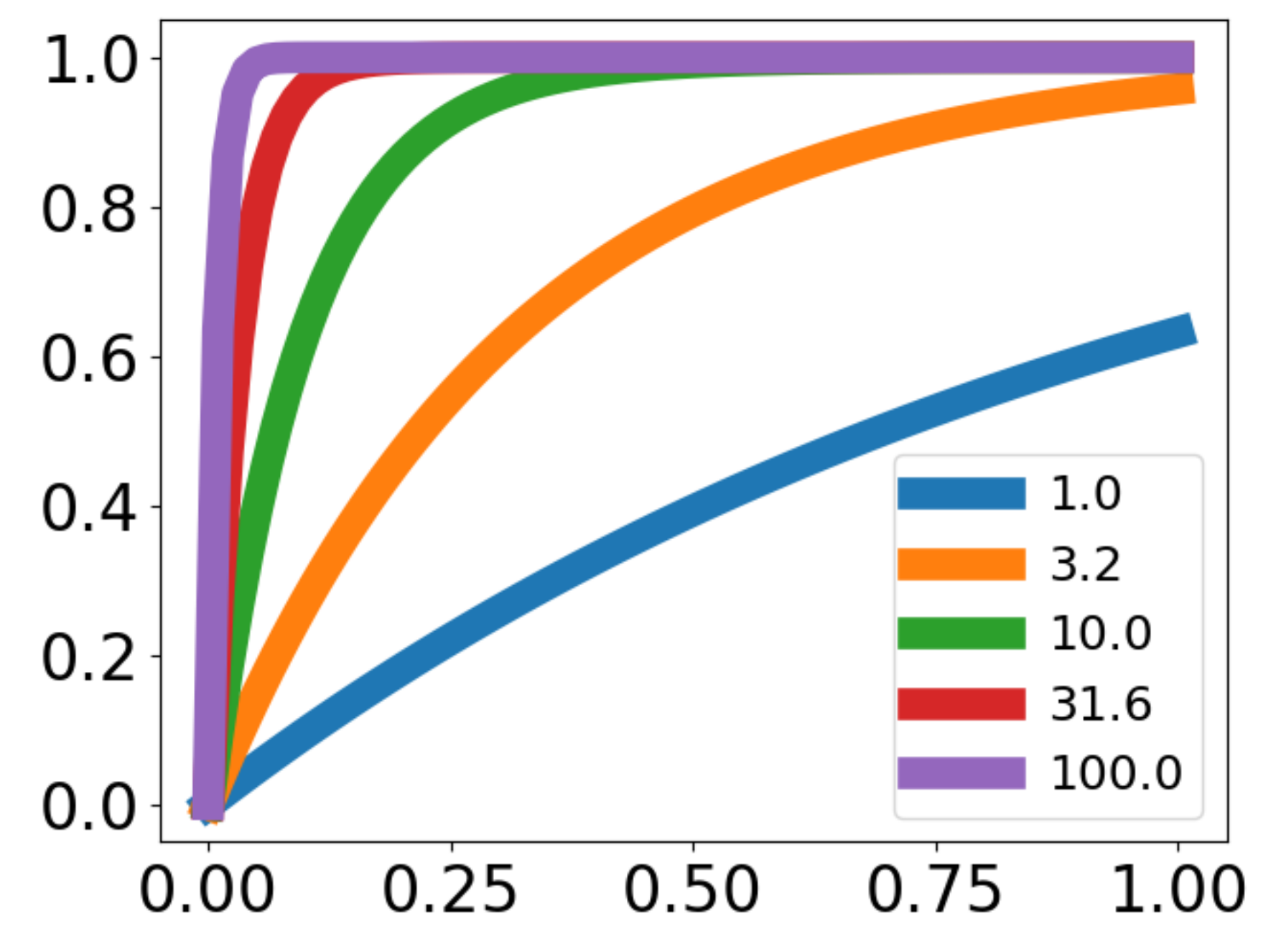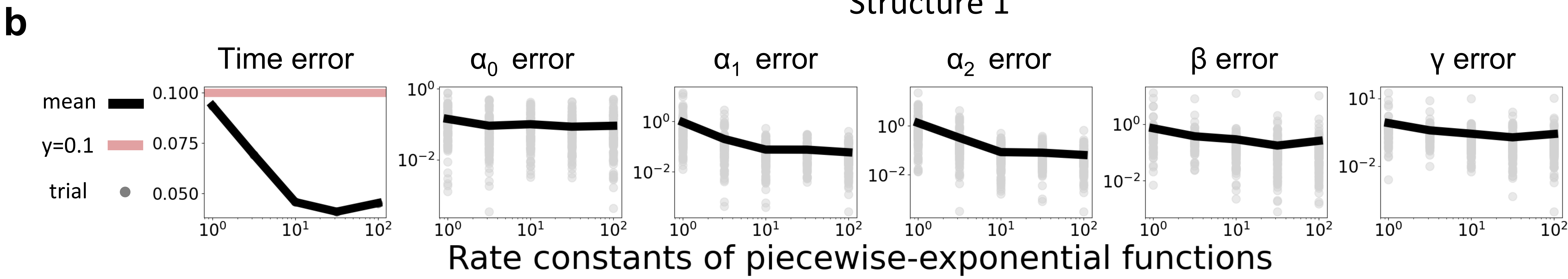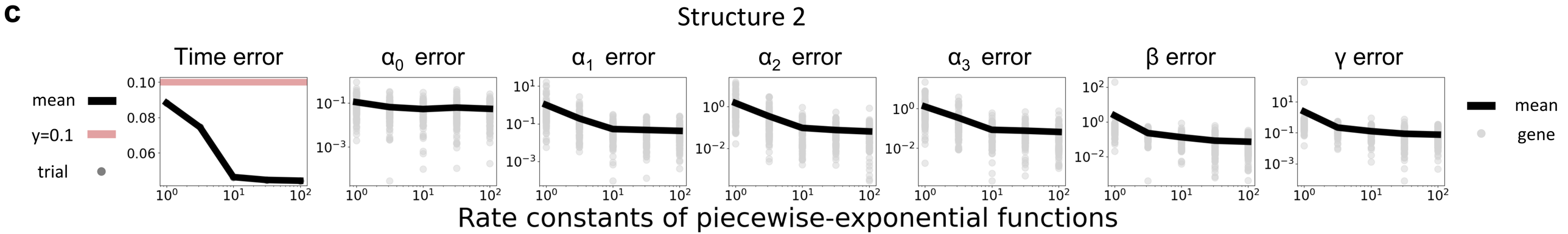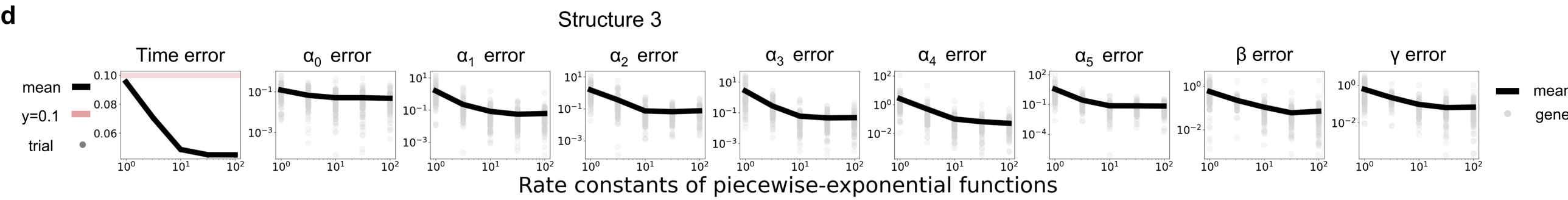

Supplement: S11 Fig — Simulations are generated using piecewise-exponential functions for transcription rates and fit under Chronocell’s piecewise-constant assumption. The three trajectory structures have been defined in S2a and S10a Figs. For time, error is root mean square error. For α , β , γ, error is mean normalized error as described in the Section Simulations. a) One piece of the piecewise-exponential functions used for transcription rates. Different rate constants are used in the simulation to span the range from an almost linear transition to an almost step function. b) Results for trajectory structure 1. c) Results for trajectory structure 2. d) Results for trajectory structure 3. (PDF) [file pcbi.1012752.s012.pdf]

# CV<sup>2</sup>-mean relationship

a

Forebrain

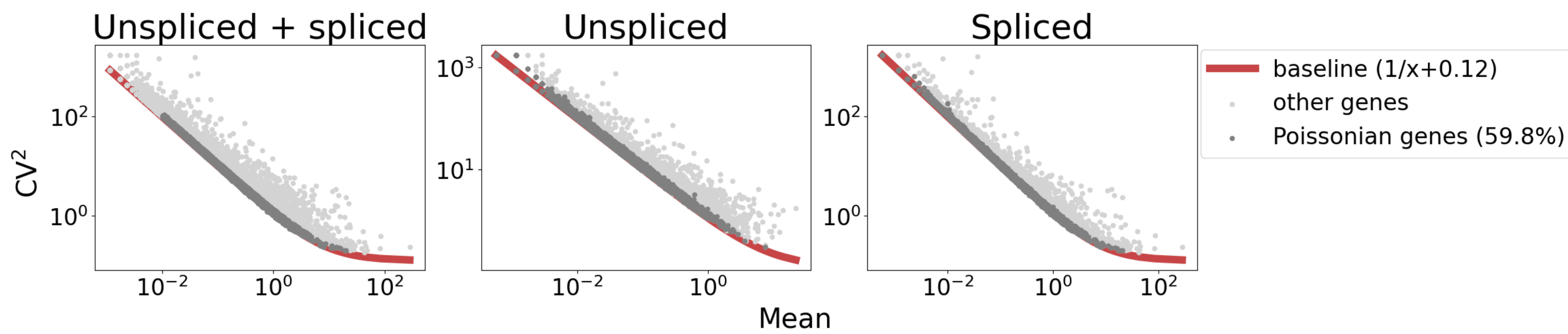

b

Erythroid

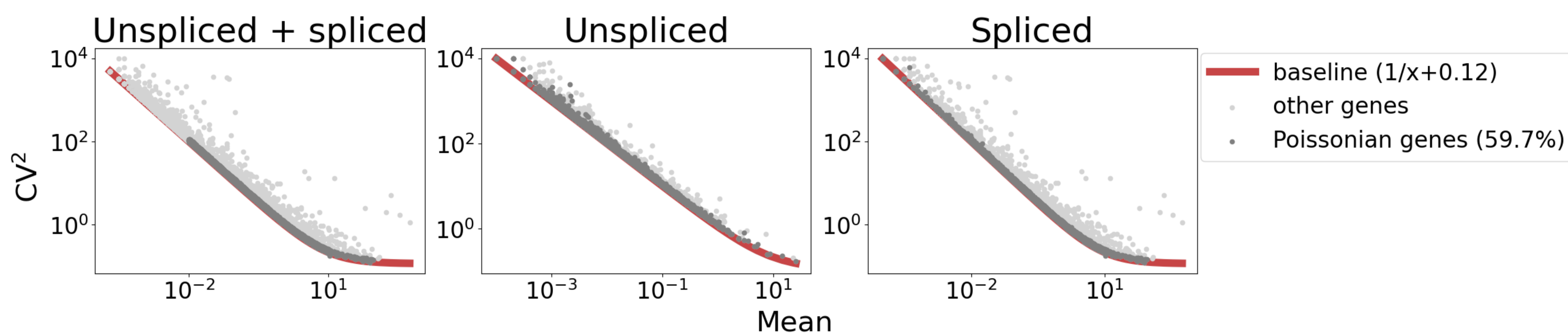

c

Cell cycle

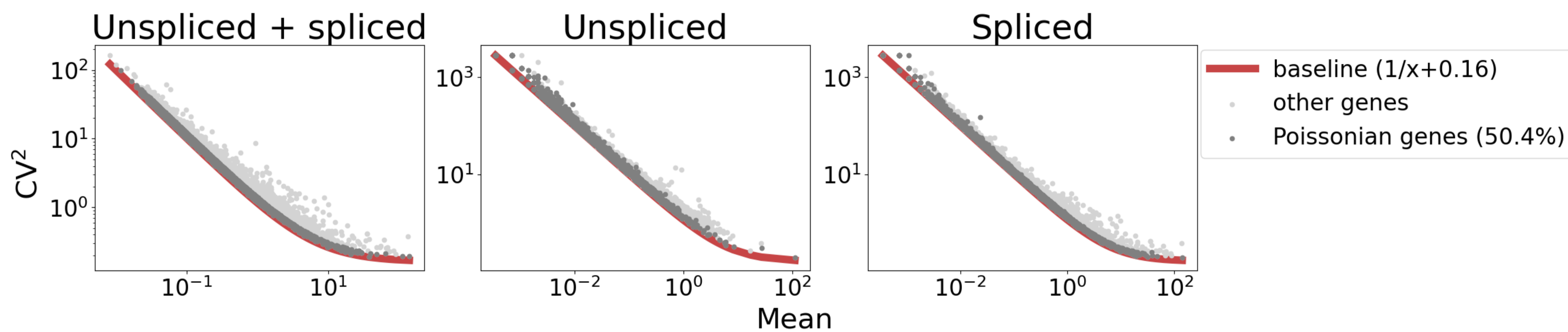

d

Neuron

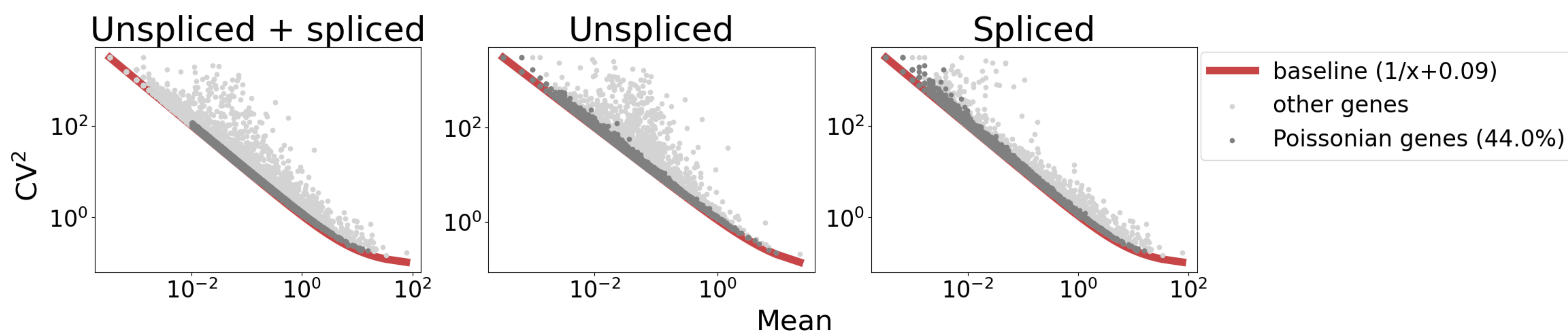

e

PBMC

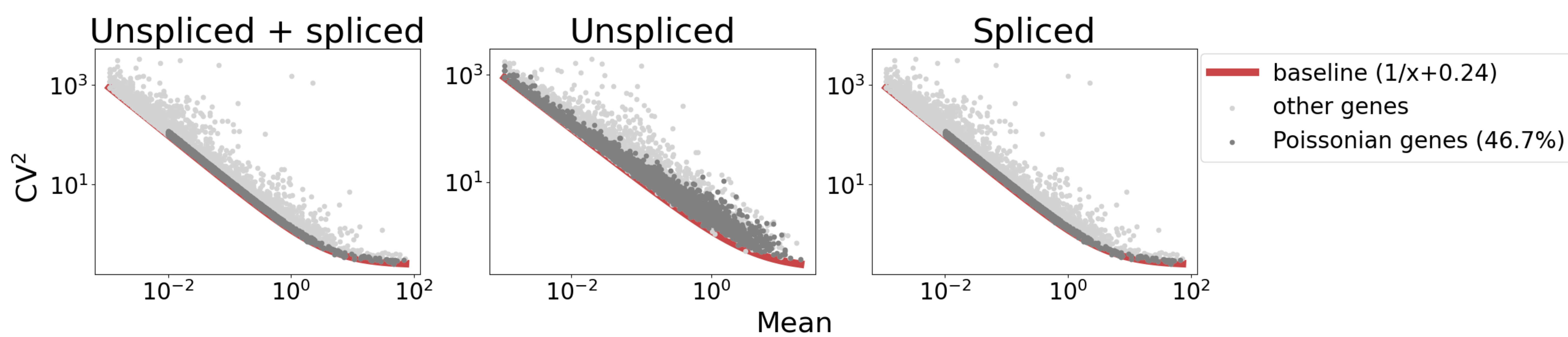

Supplement: S16 Fig — a) Forebrain data. b) Erythroid data. c) Cell cycle data. d) Neuron data. e) PBMC data. (PDF) [file pcbi.1012752.s017.pdf]

## a Read depth estimates

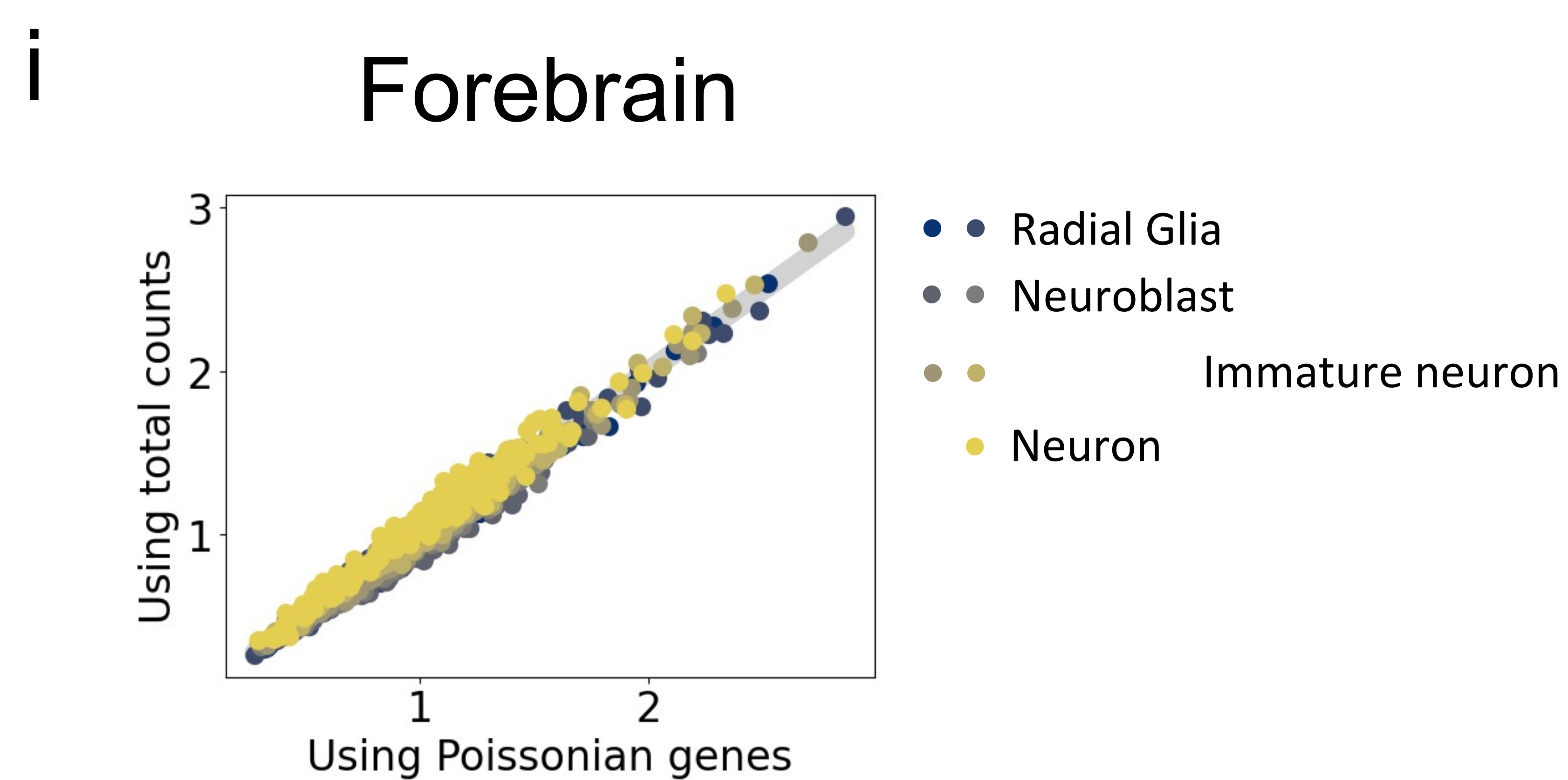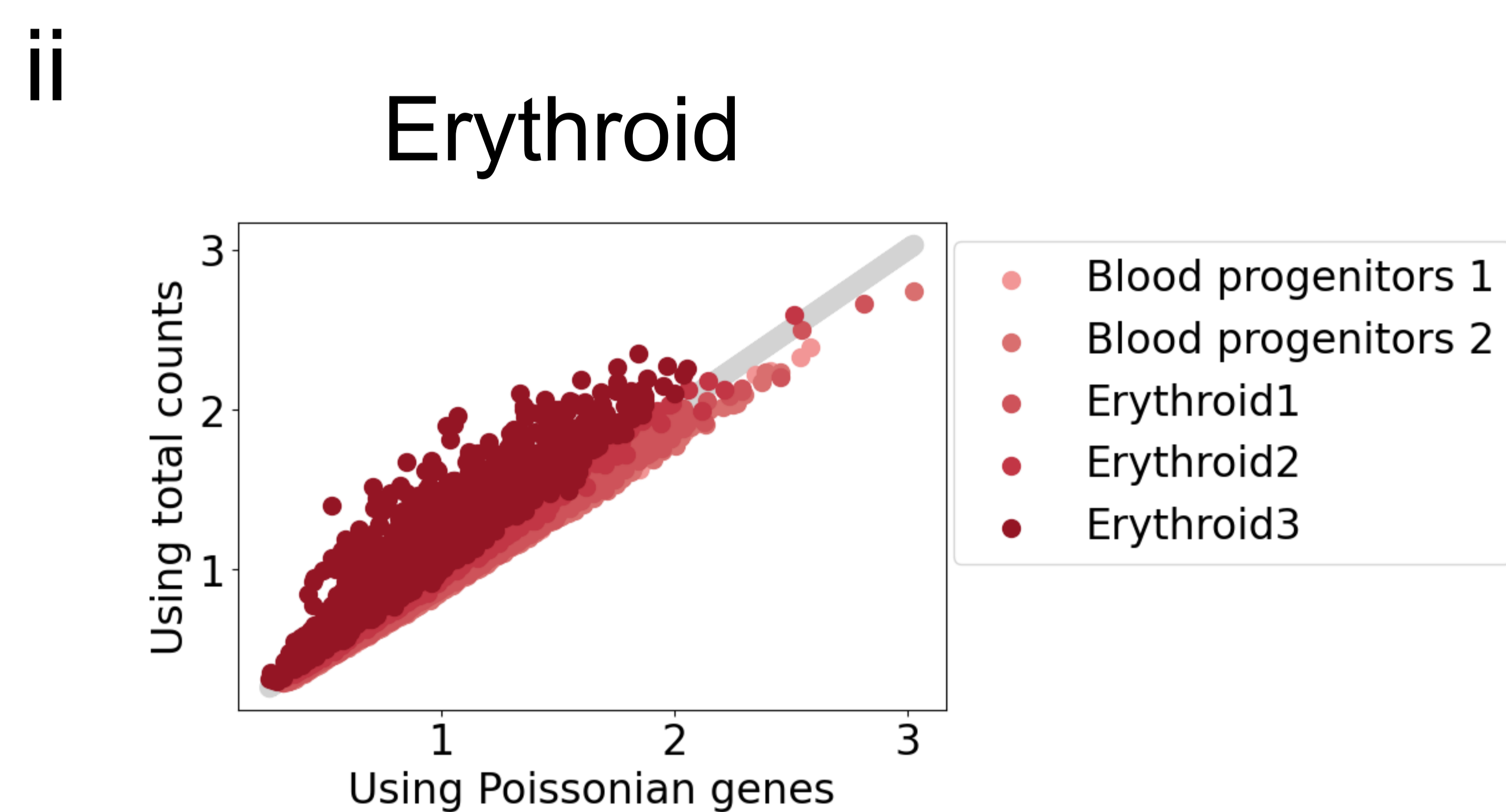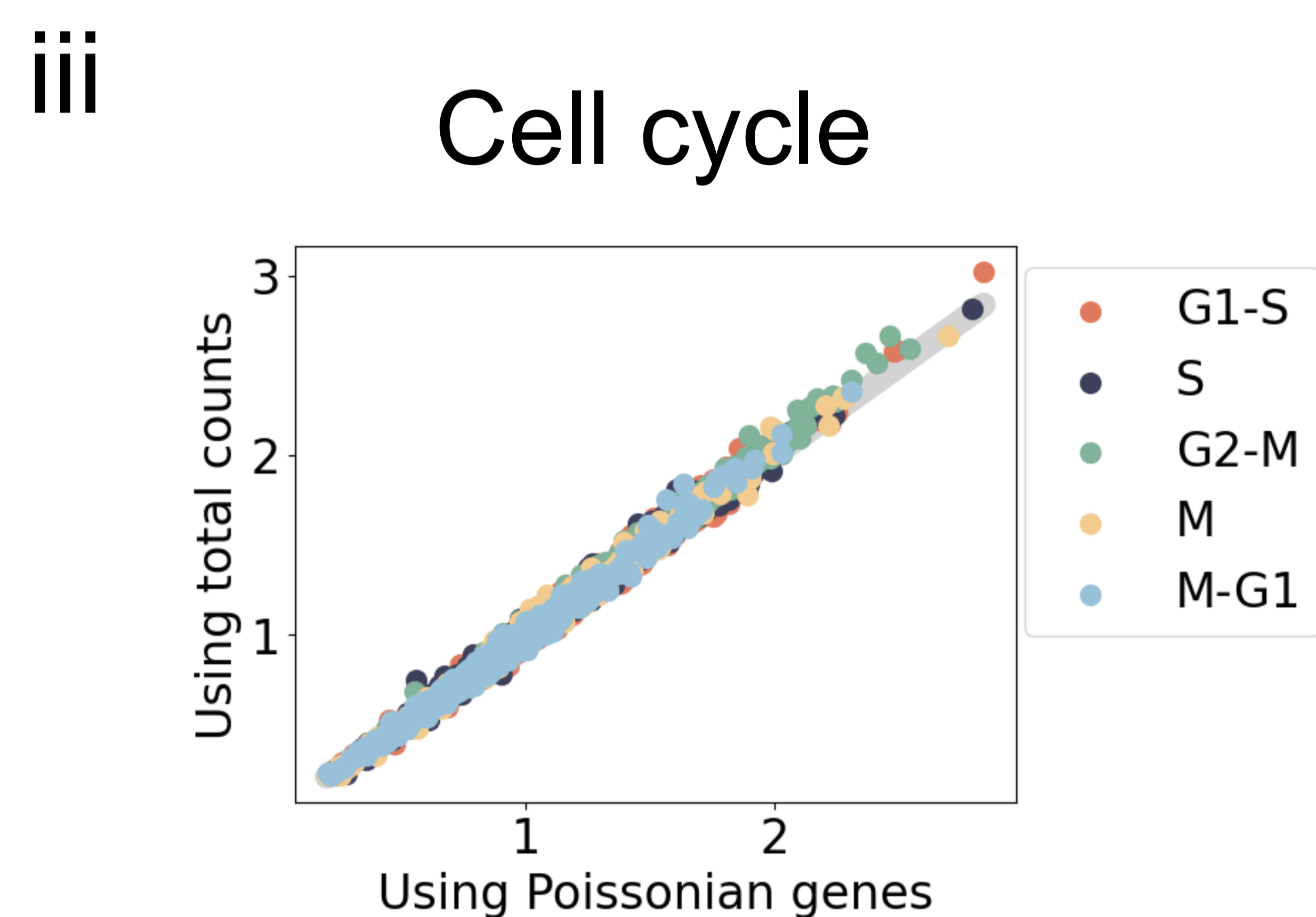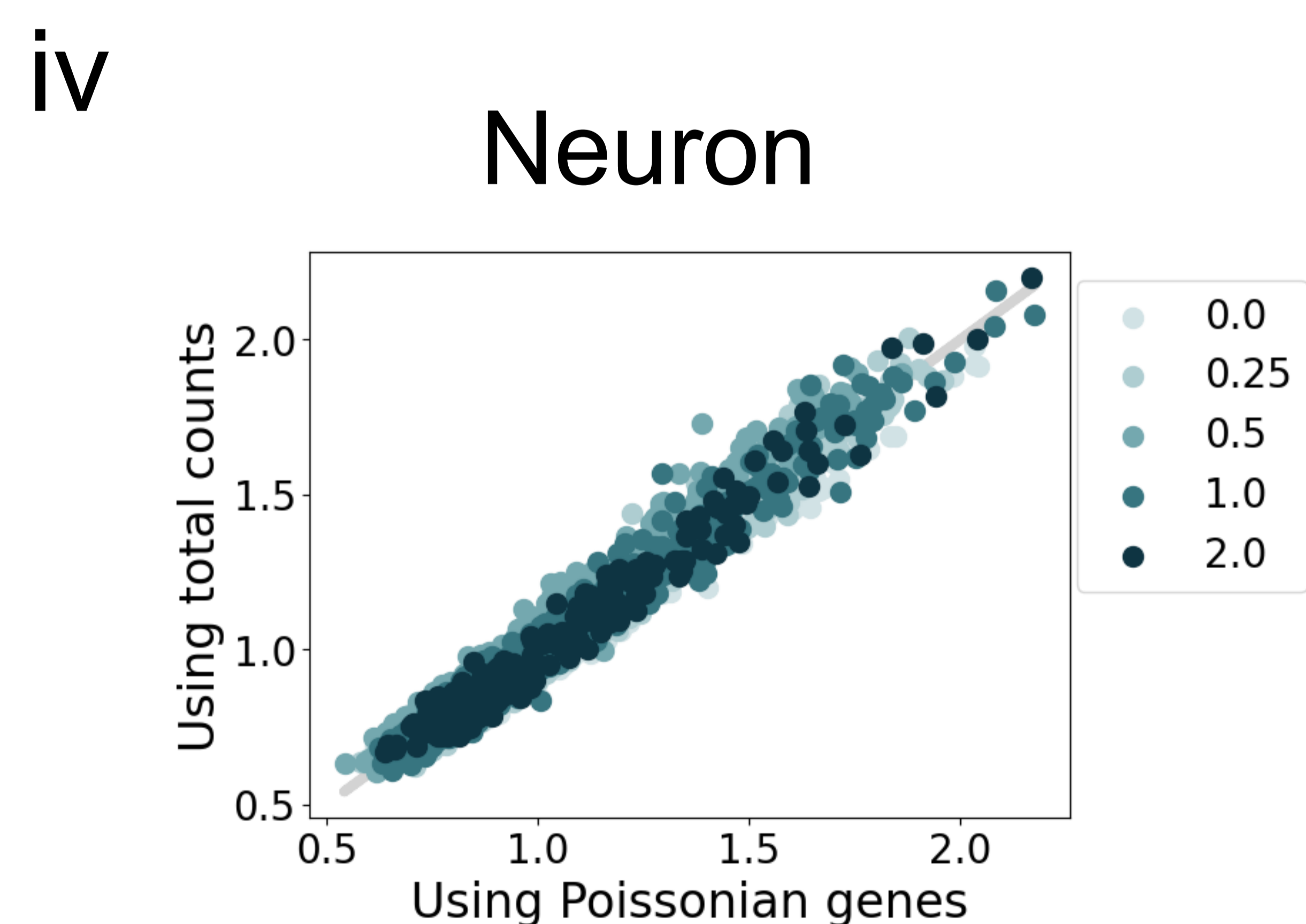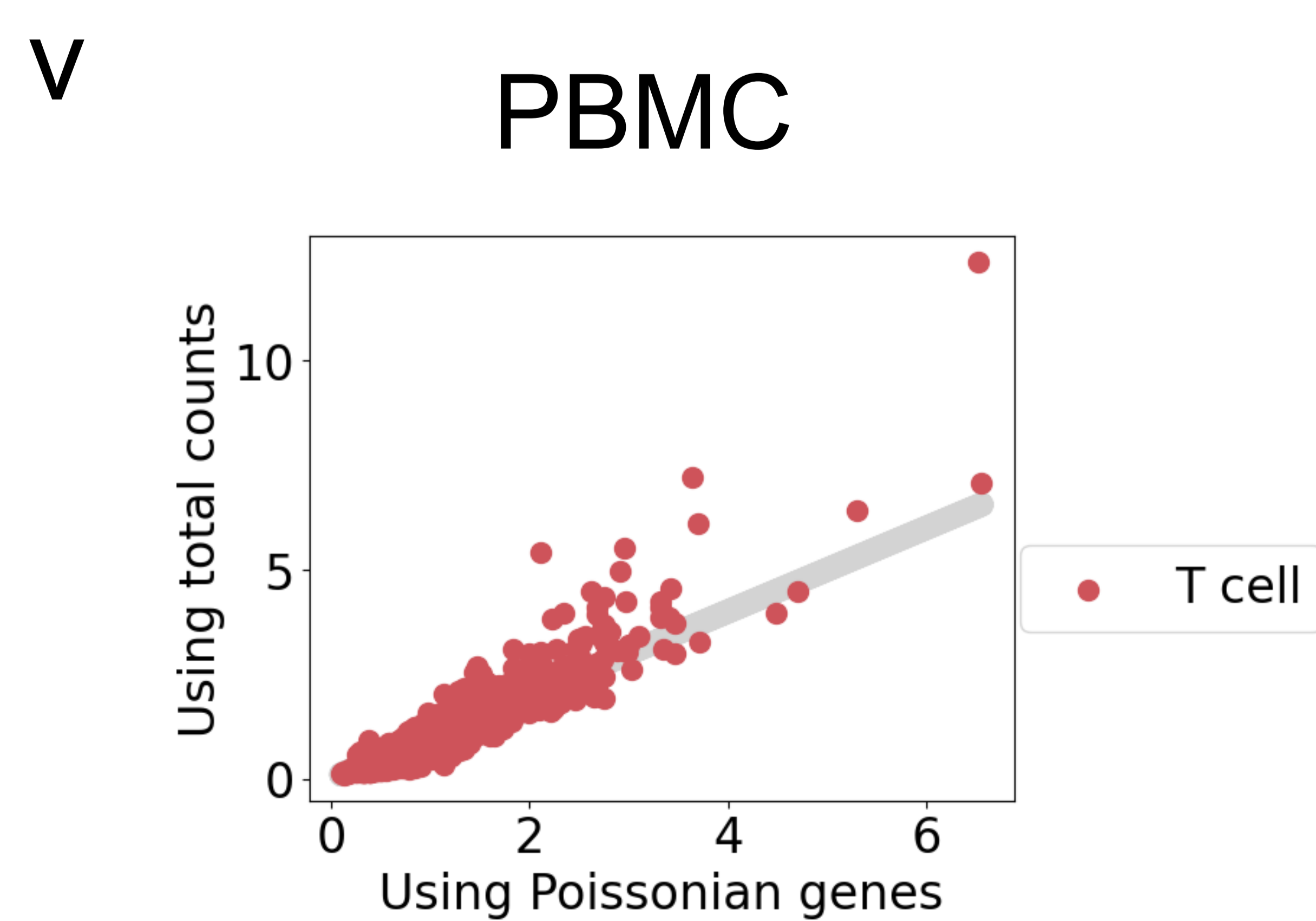

## b Filtered genes for fitting

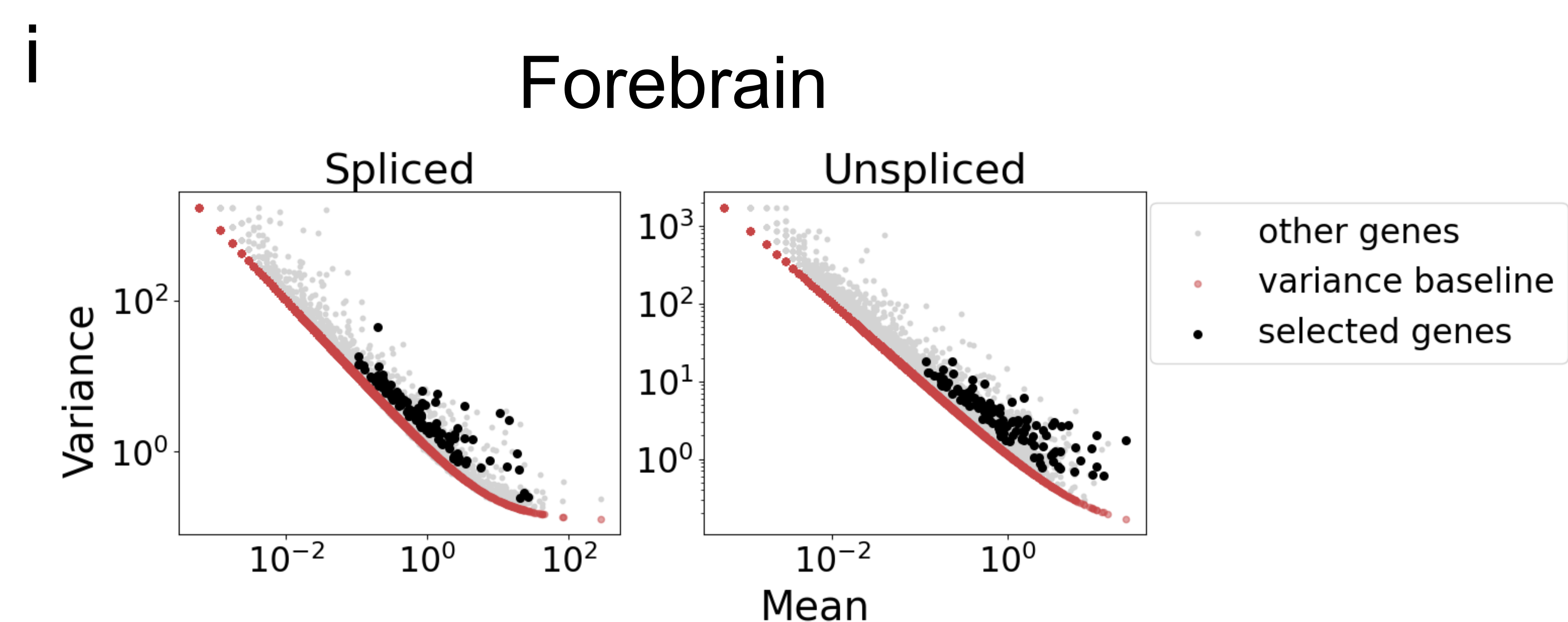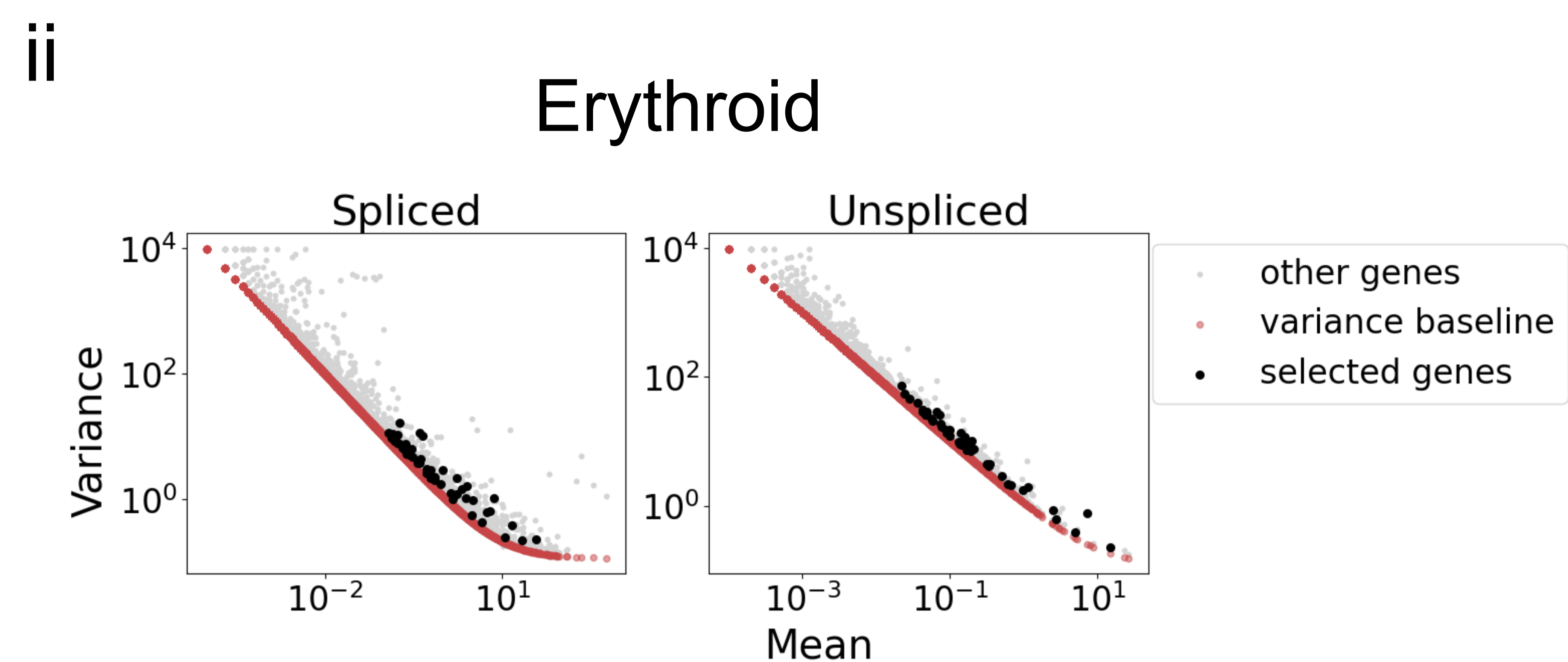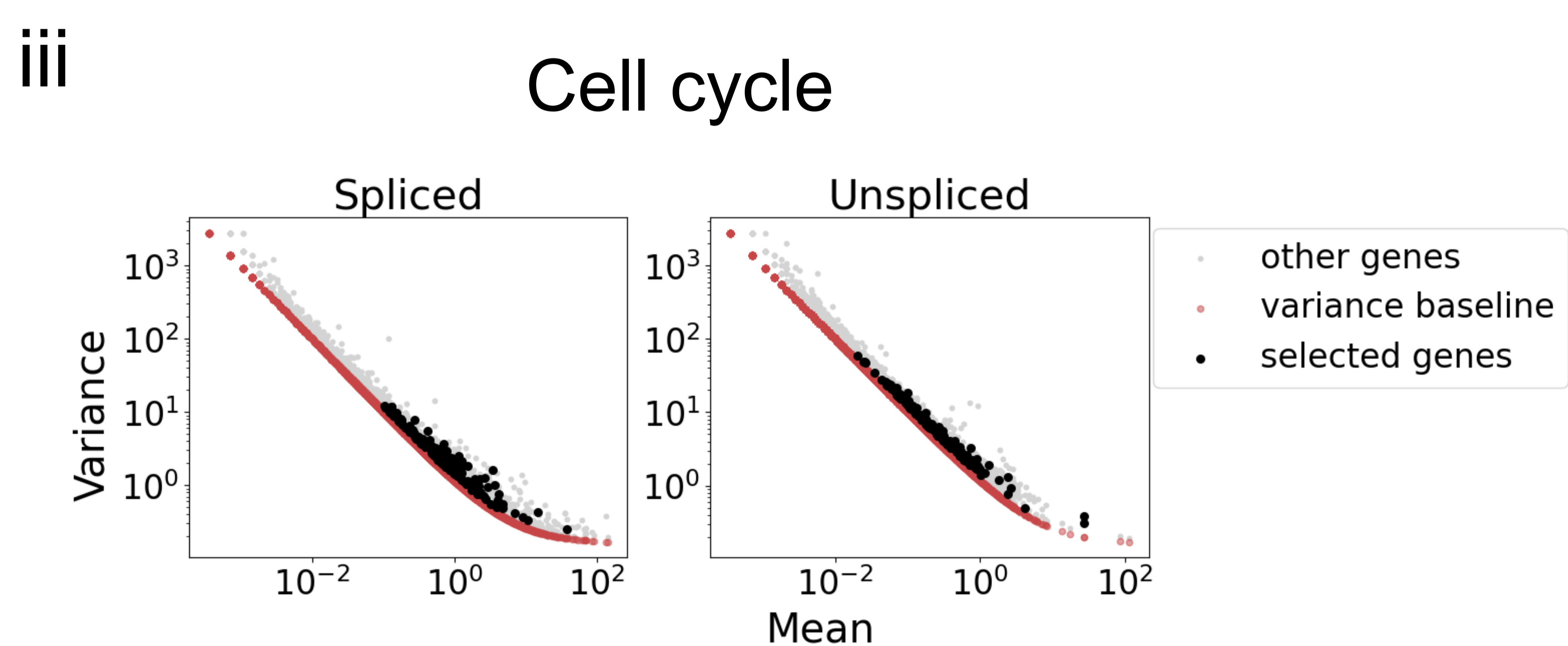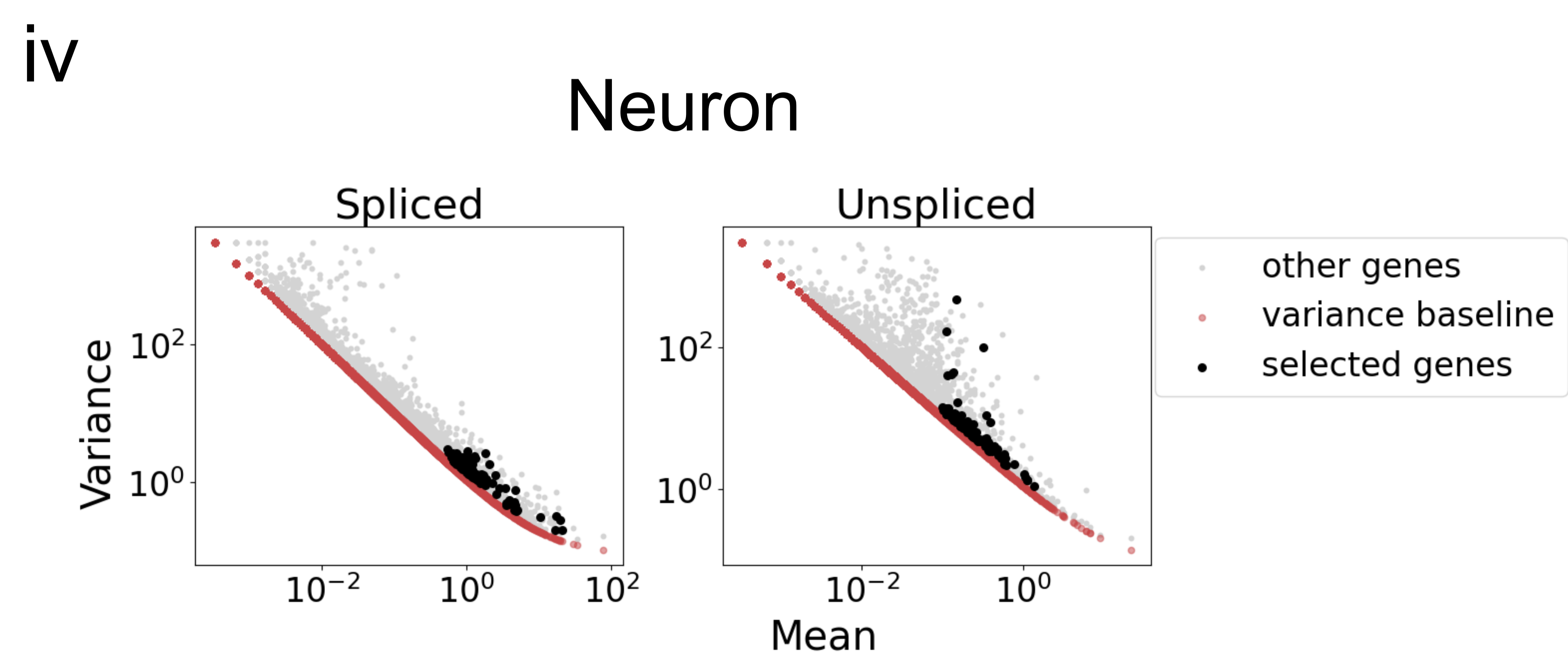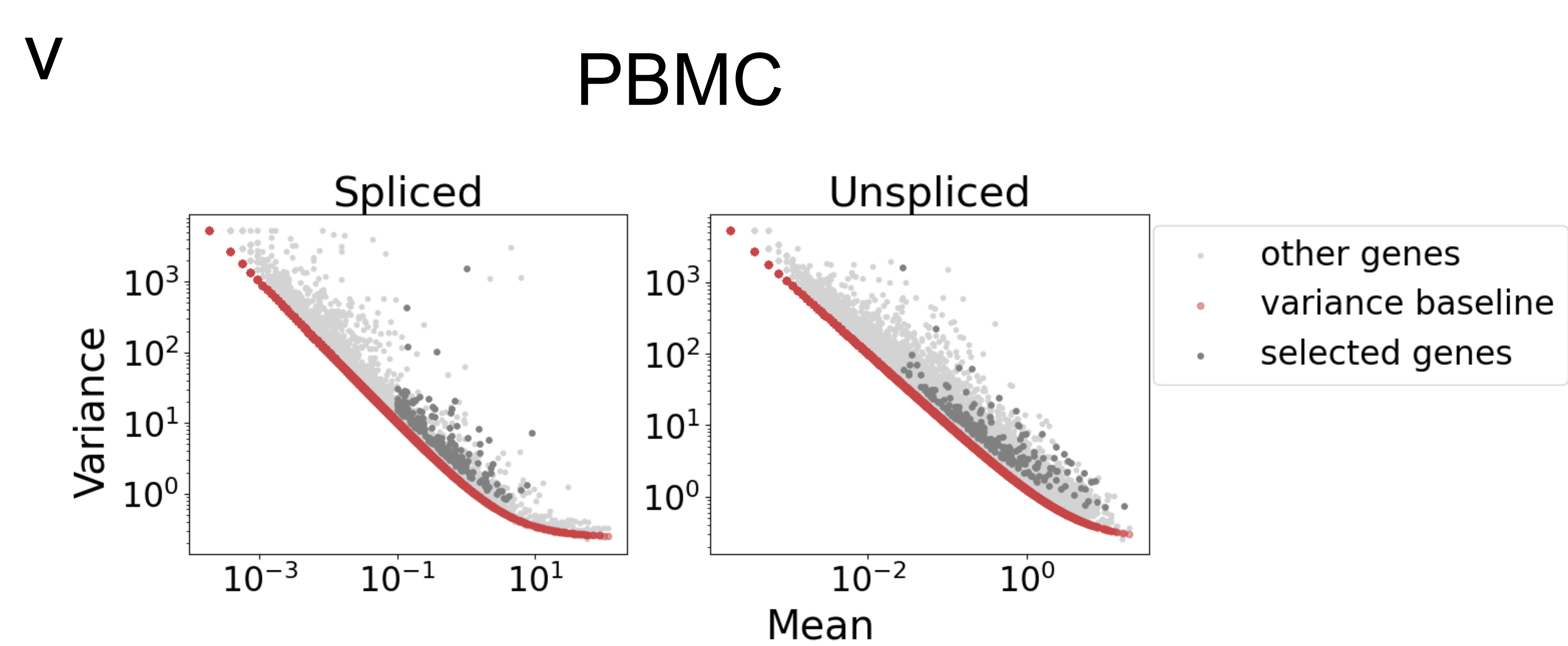

Supplement: S17 Fig — a) Read depth estimation based on total counts of Poissonion genes. Cells are colored by their cell types. b) Selected genes (black) for fitting plotted in the same CV2-mean plot as in S16 Fig. (PDF) [file pcbi.1012752.s018.pdf]

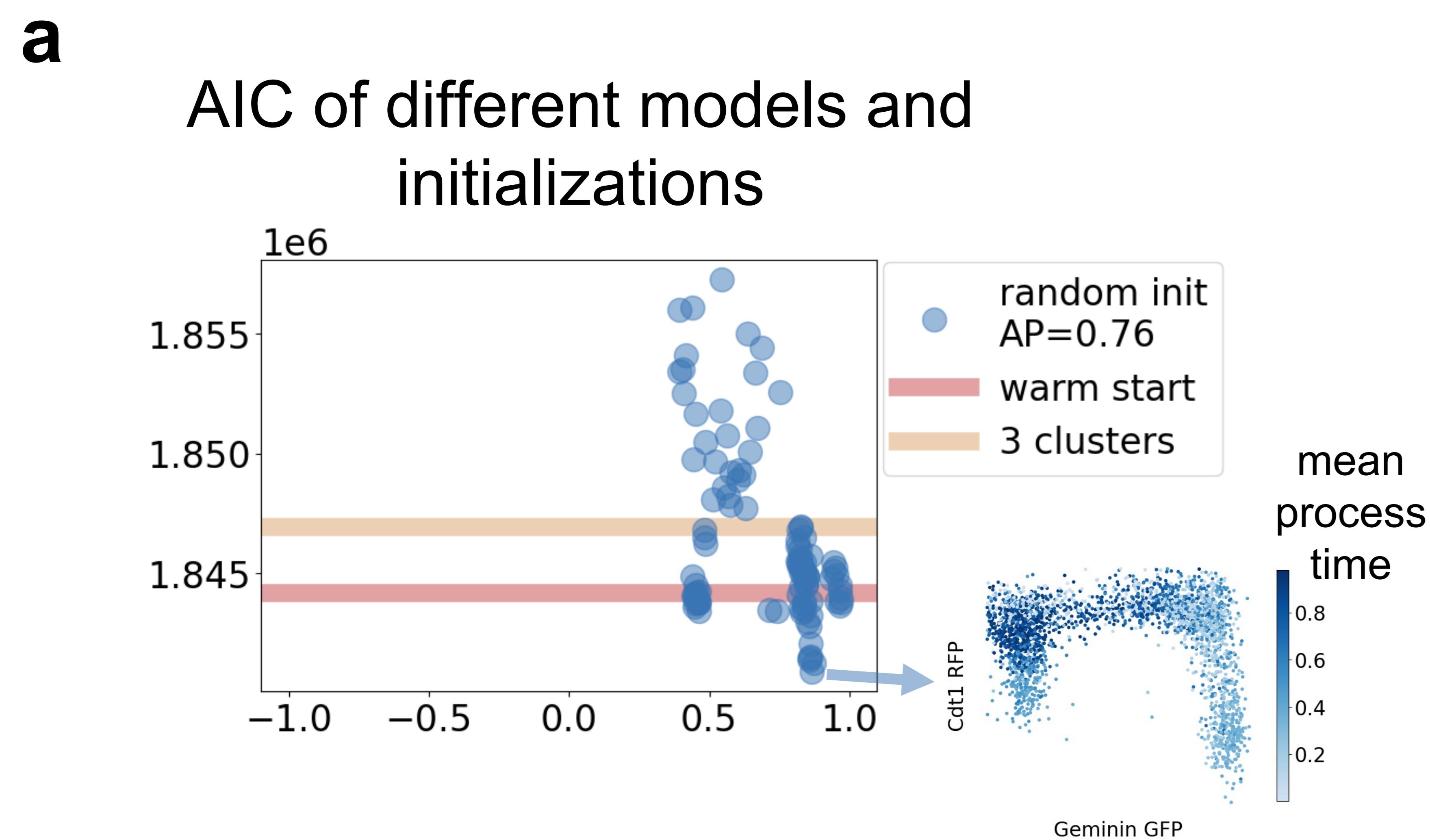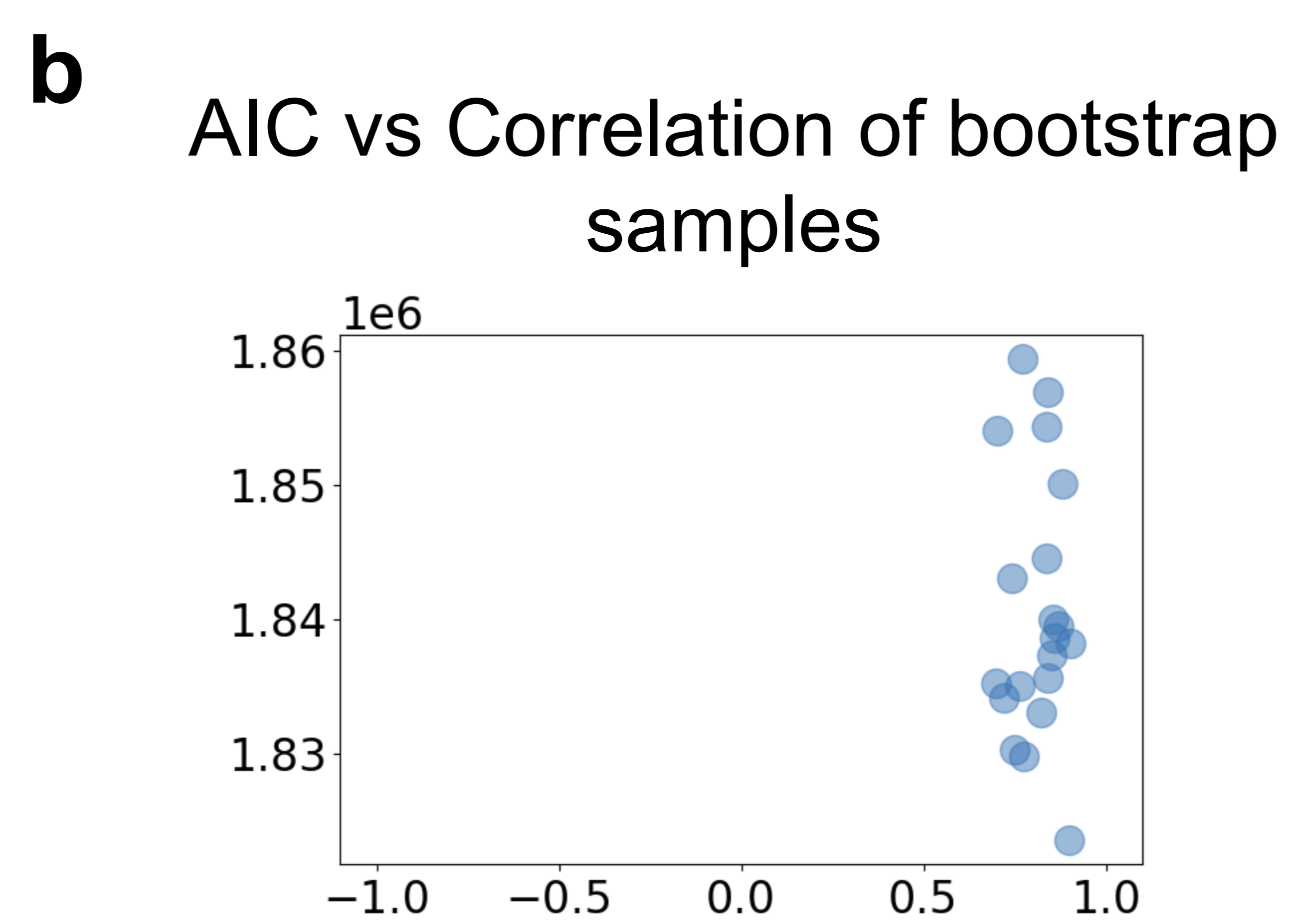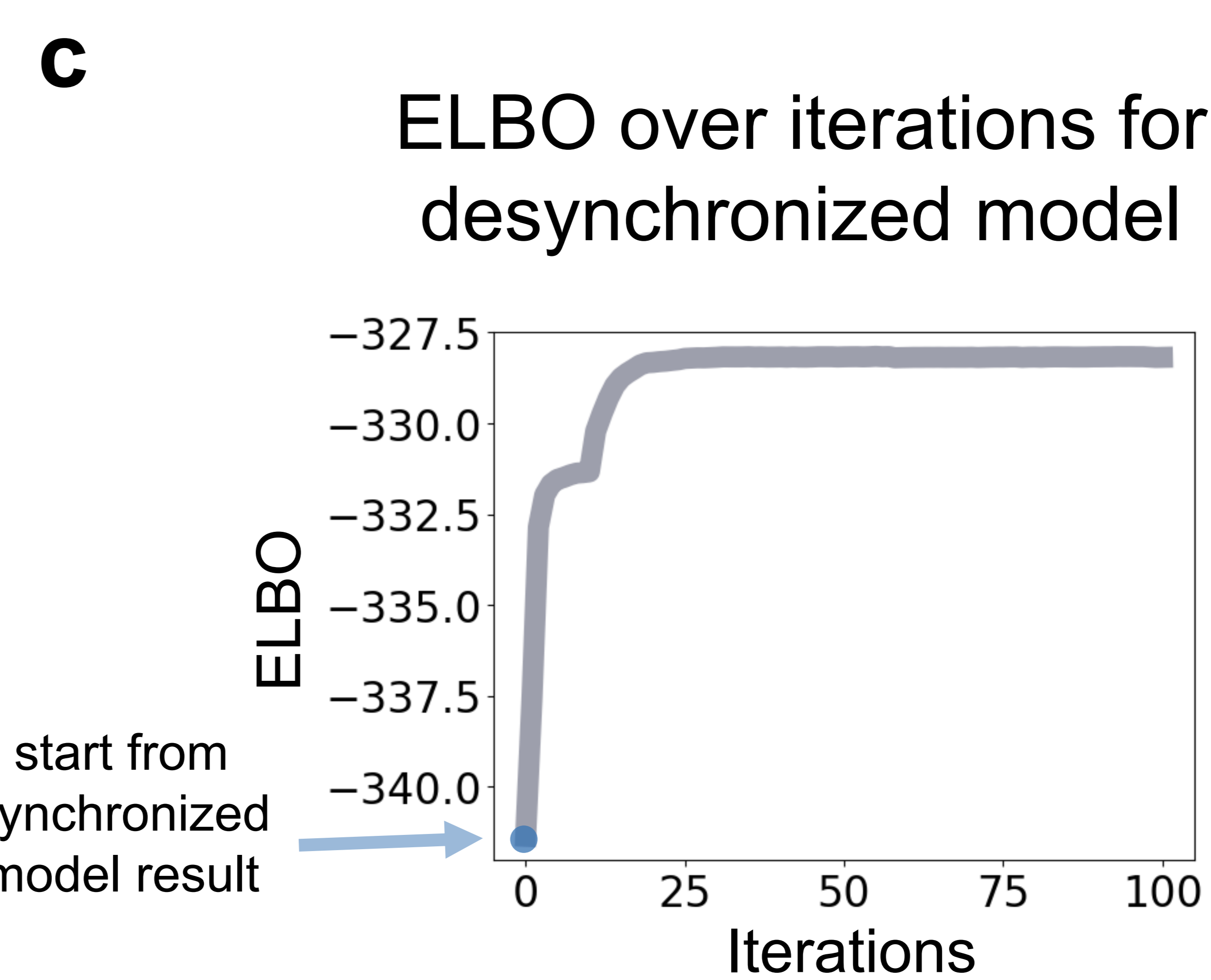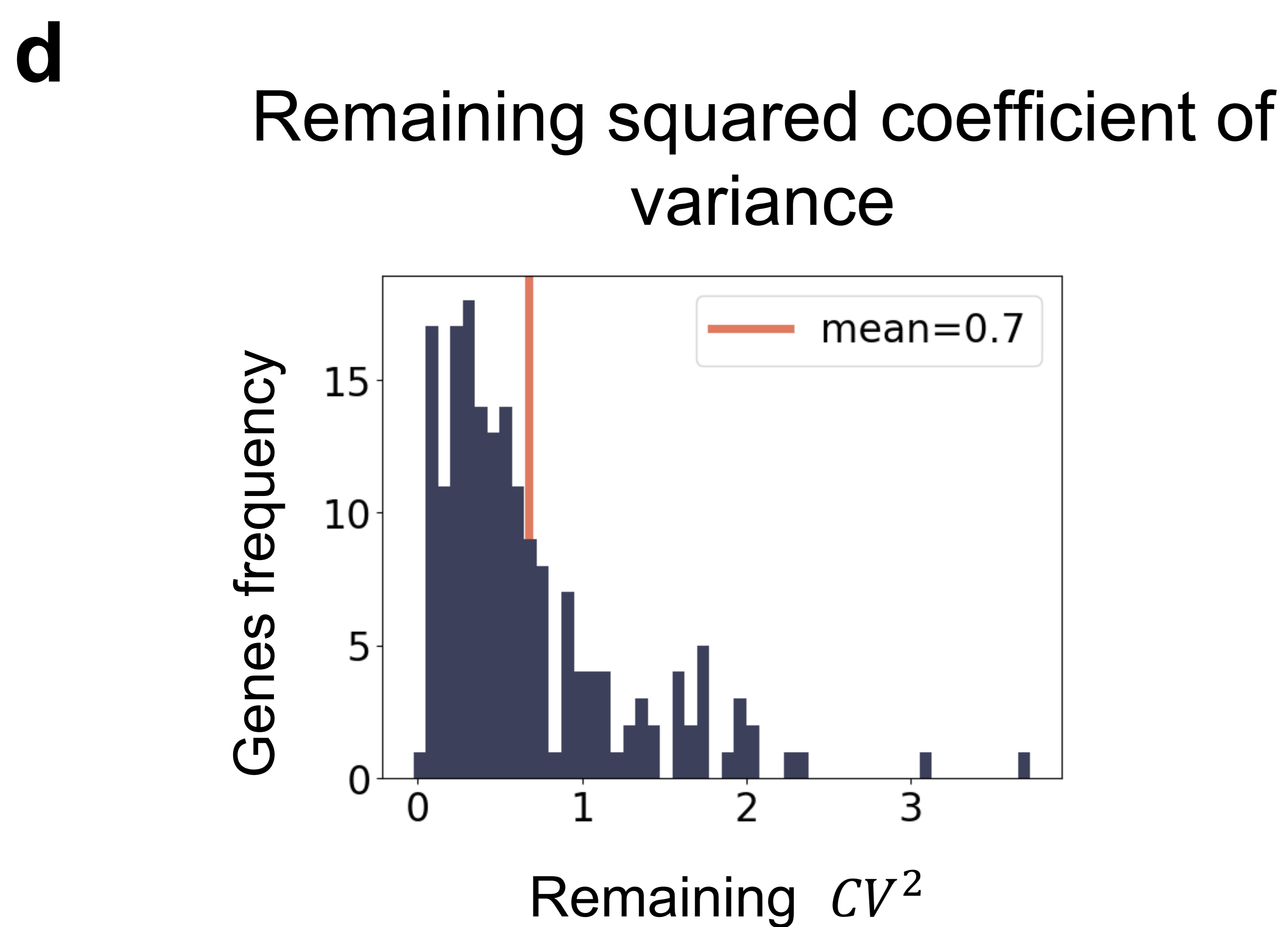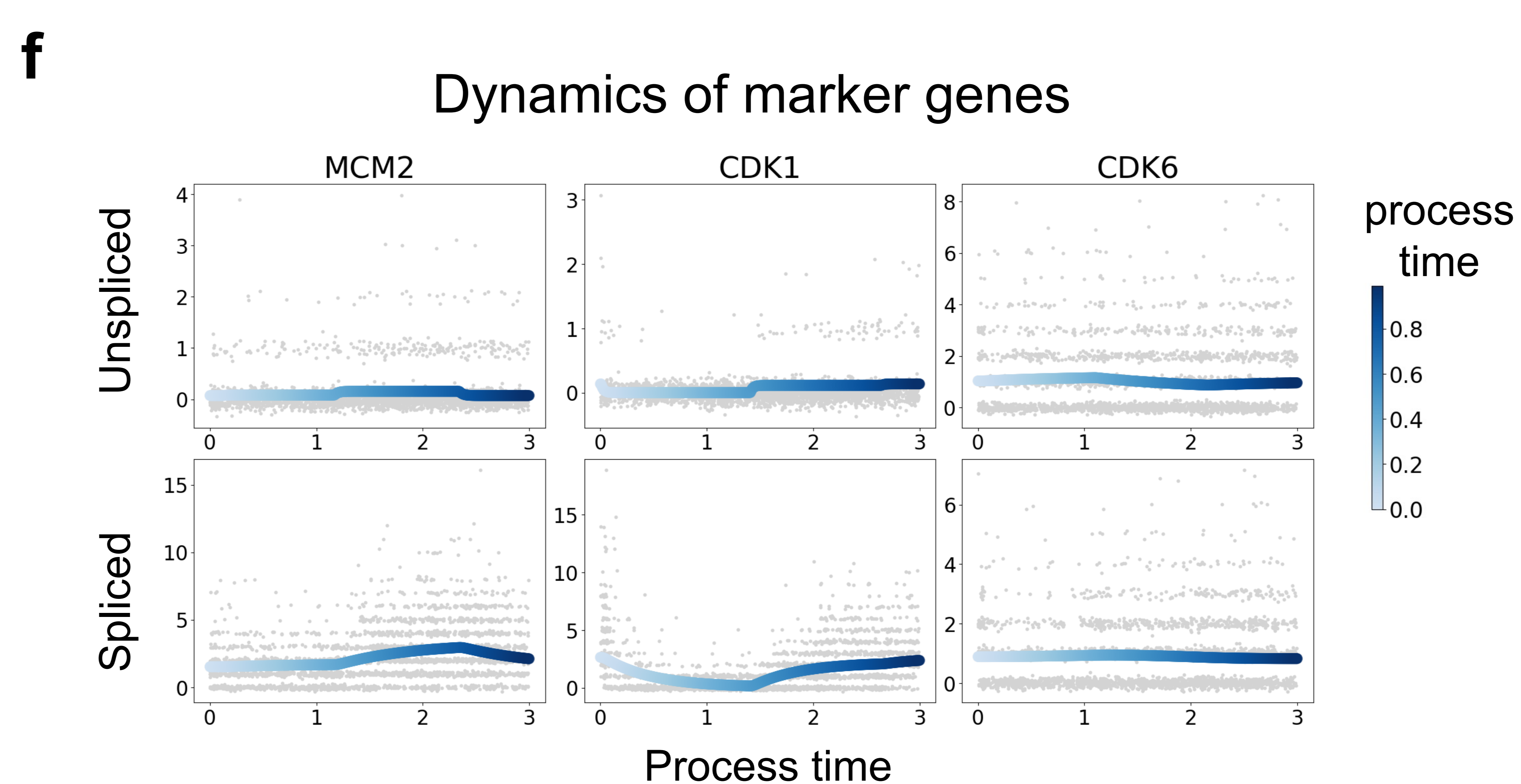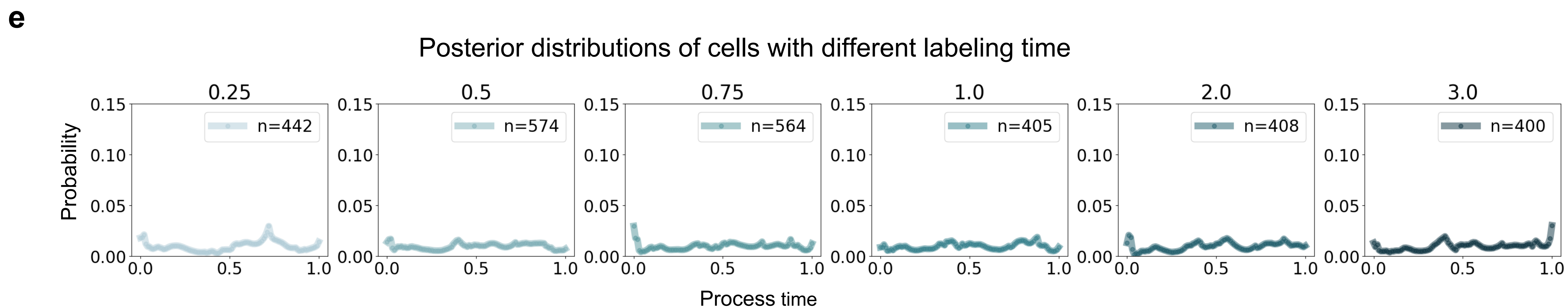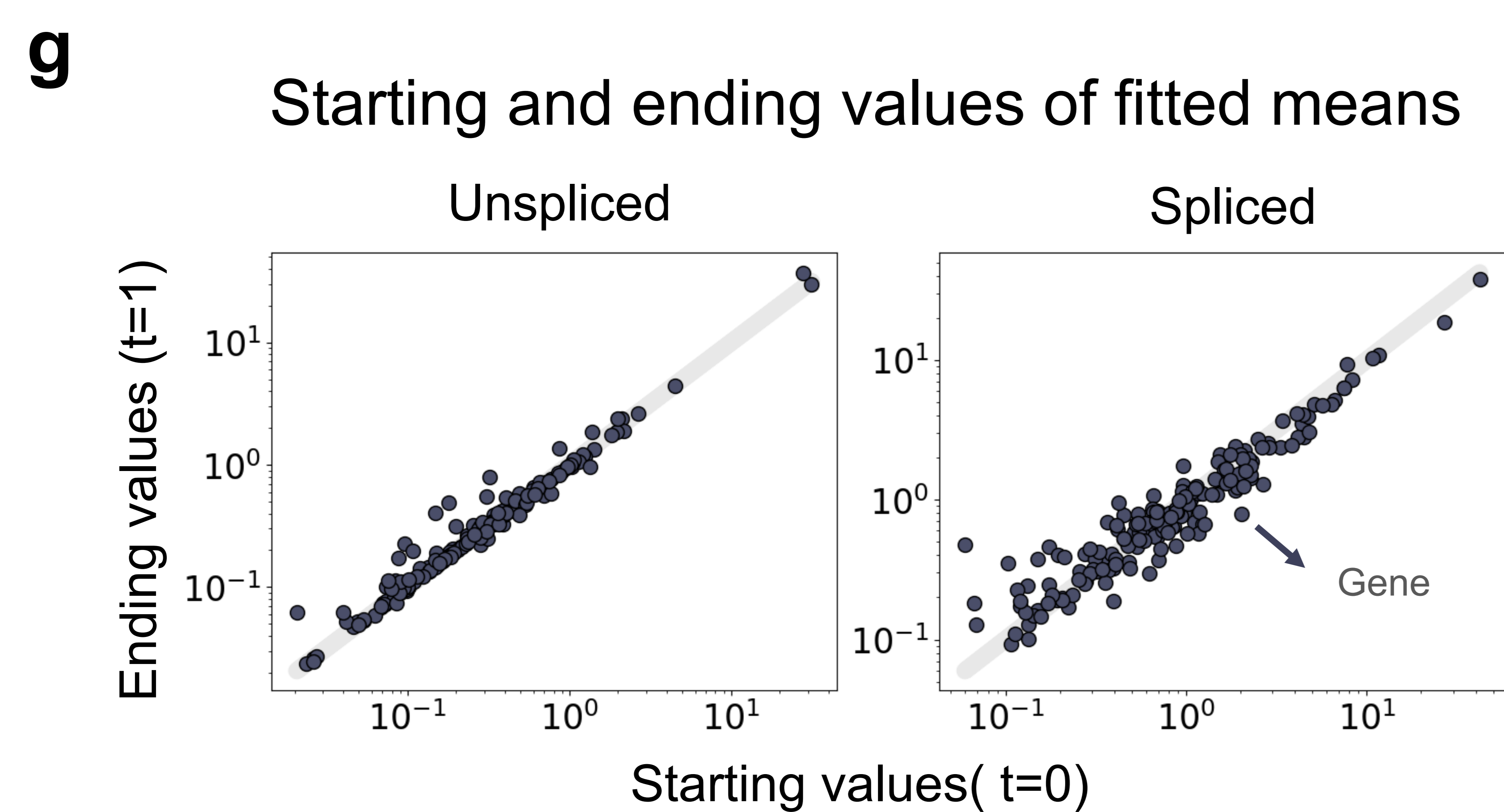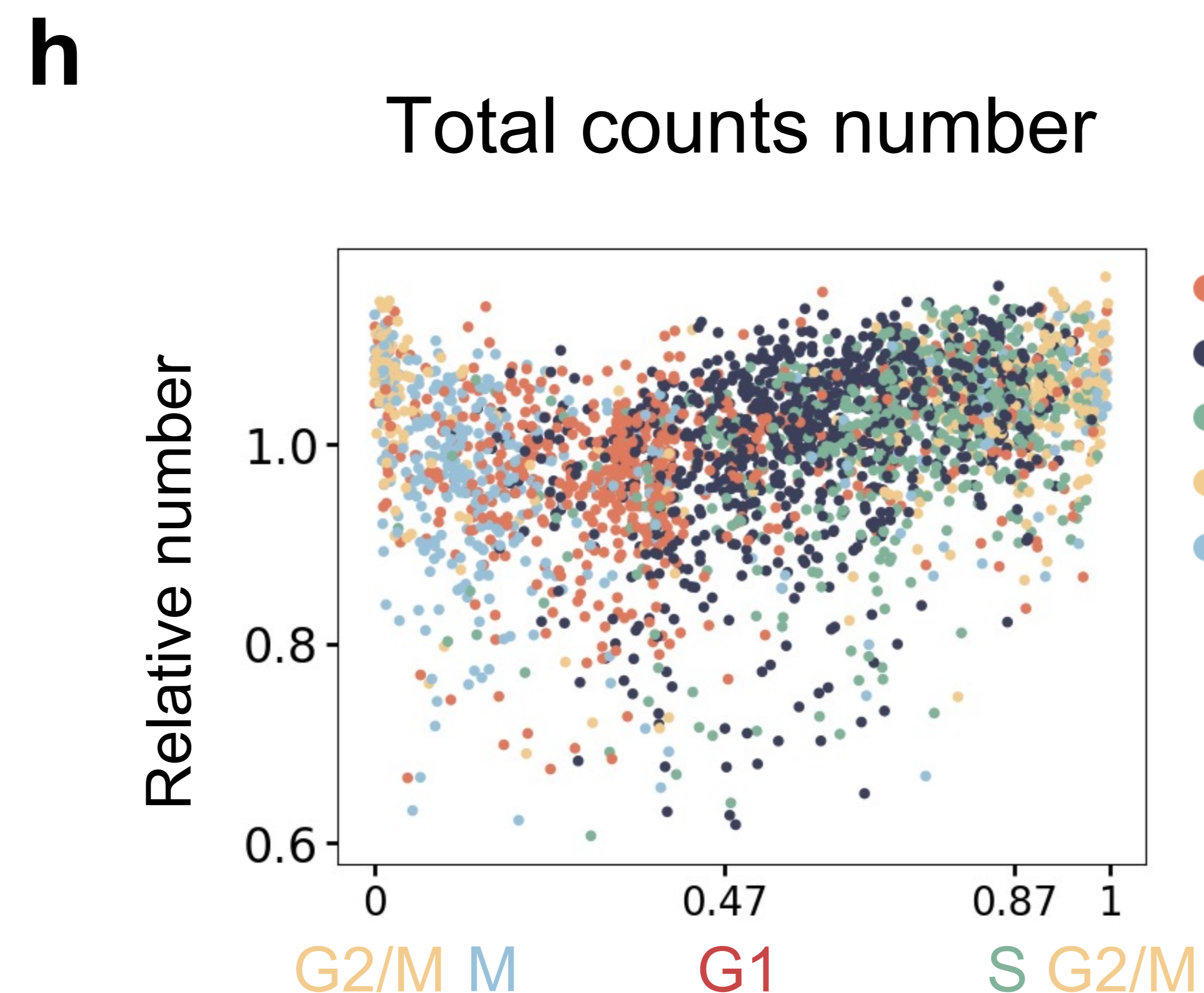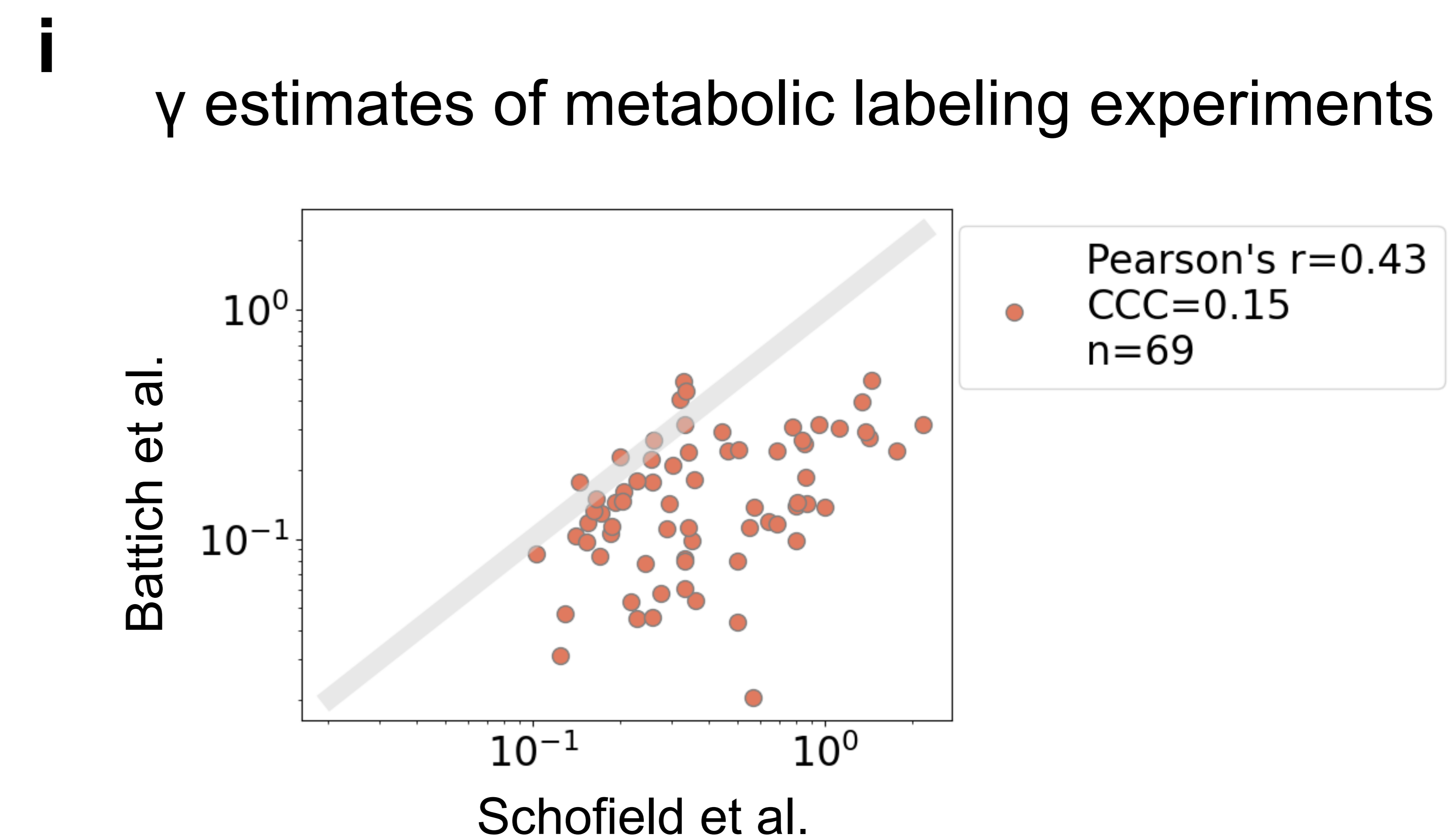

Supplement: S20 Fig — a) AIC scores and mean process time correlations of 100 random initializations (blue dots) compared to those of warm start (red line) as well as 3 clusters (Poisson mixtures) model (yellow line). AP stands for average precision. Mean process time of the initialization with lowest AIC is indicated in blue on the same PCA plot as in a. b) AIC scores and mean process time correlations of 100 bootstrap samples. The x axis is the Pearson’s correlation between the mean process time of each bootstrap and the those of original data, i.e., the plotted one in a. c) ELBO scores over iterations for desynchronized model. The fitting started with the best random initializations result of synchronized model. d) Distribution of remaining squared coefficient of variance of 182 genes used in the fitting. Remaining squared coefficient of variance is calculated by dividing the remaining unexplained variance by mean squared. e) Averaged posterior distribution across cells with different labeling times. n is the number of cells. f) Dynamics of three marker genes. The blue curve is the fit mean of product Poisson distributions of unspliced and spliced counts over process time, and its darkness corresponds to the value of process time. Cells’ raw counts (gray) are plotted against their corresponding process times. g) Starting and ending values of fit mean of Poisson distributions. h) Total counts over process time of cells colored by cell type annotations. i) Comparison of γ estimates from two metabolic RNA labeling papers for 84 selected genes. Estimates of 67 genes are available in both papers. CCC stands for concordance correlation coefficient. (PDF) [file pcbi.1012752.s021.pdf]

**a**

Monocle 3

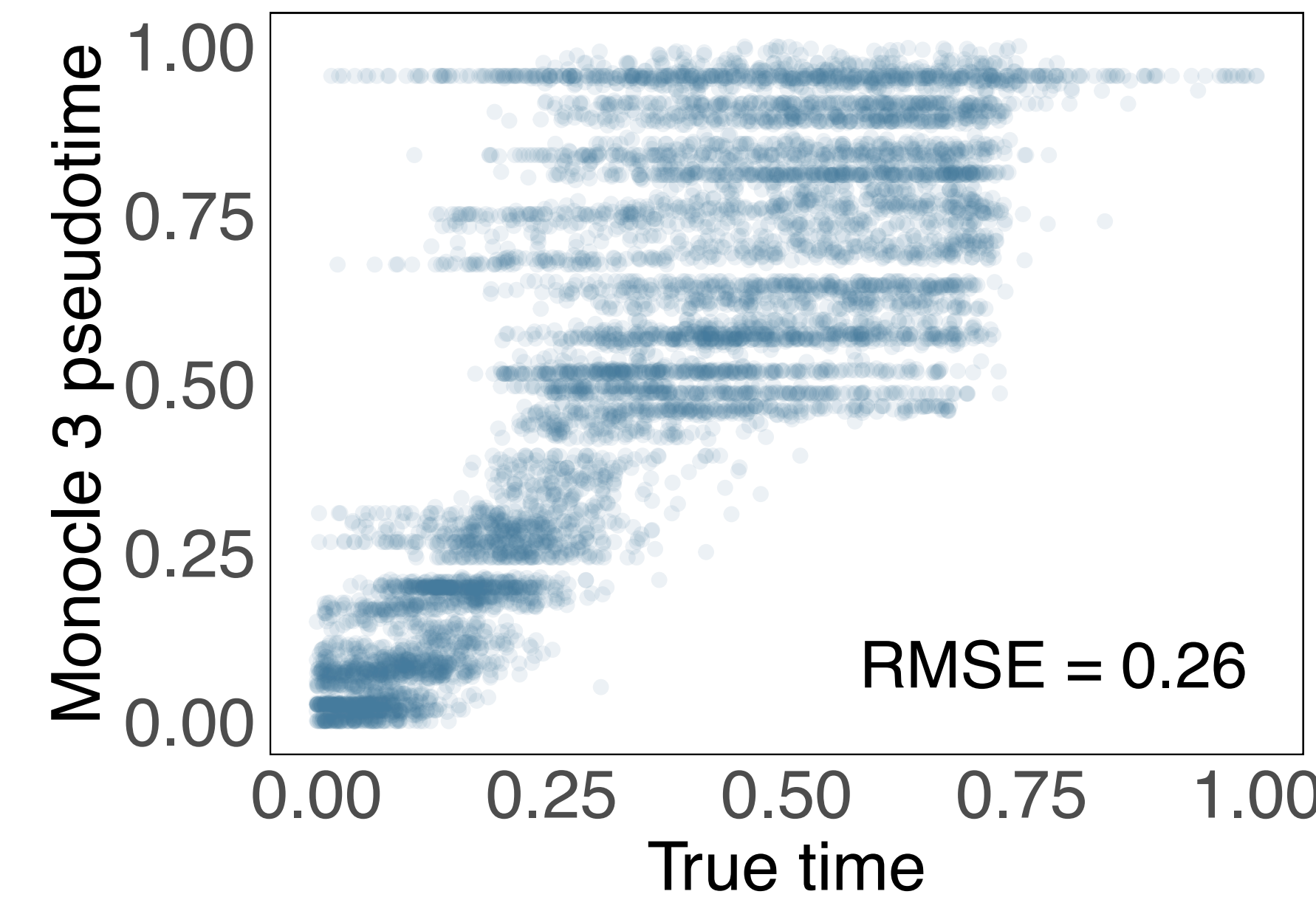**b**

Slingshot

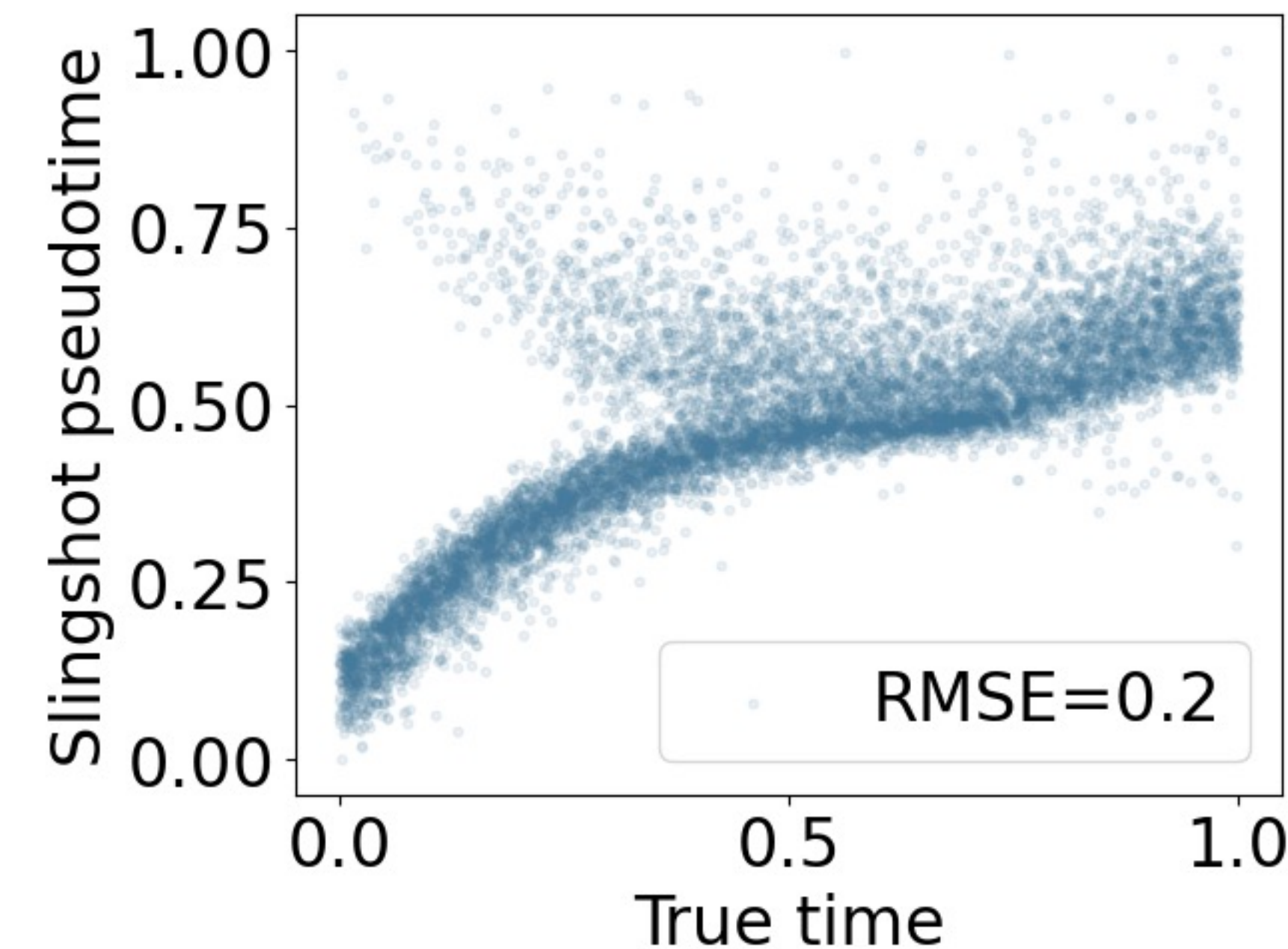**c**

Diffusion pseudotime

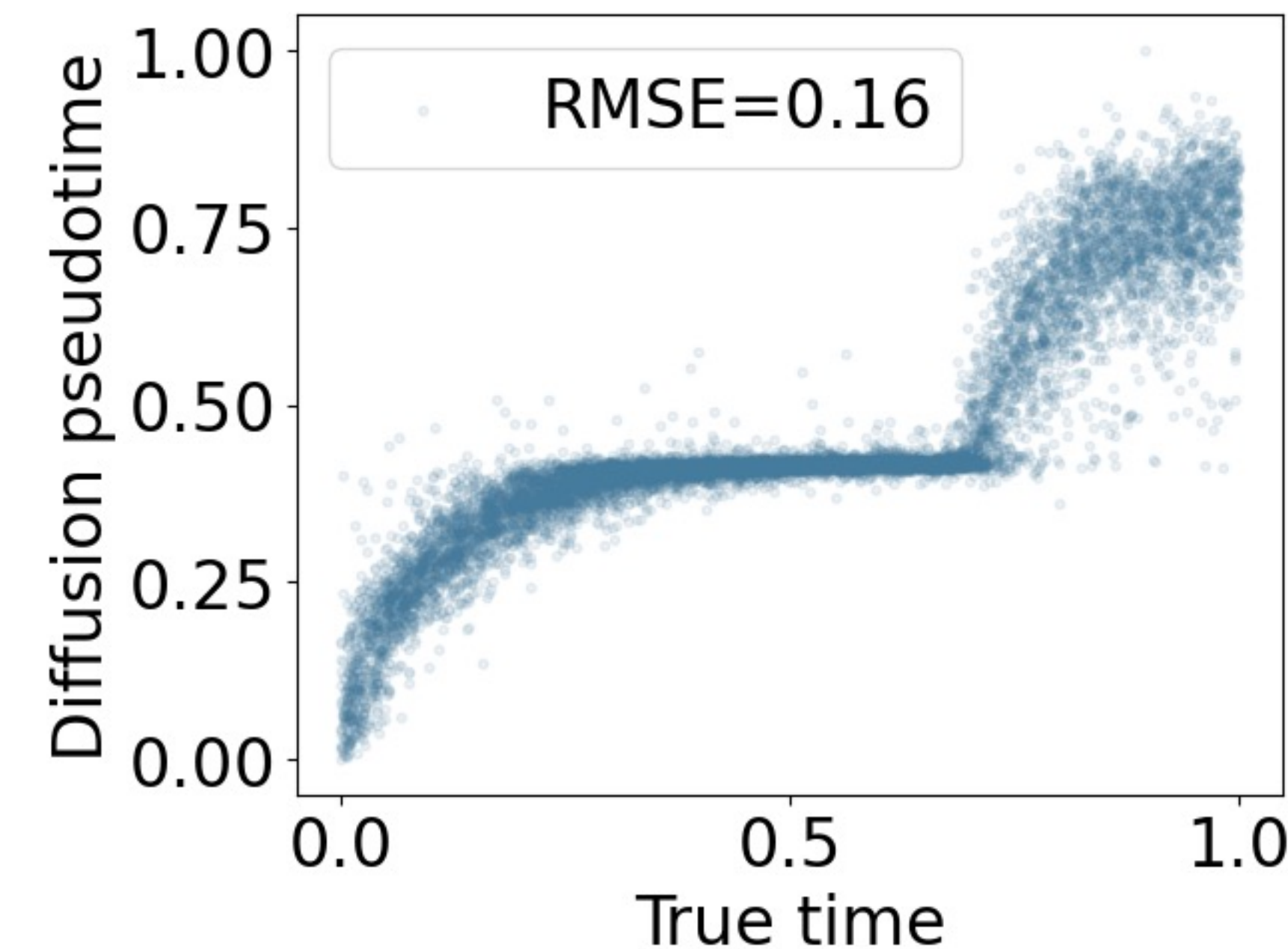**d**

veloVI

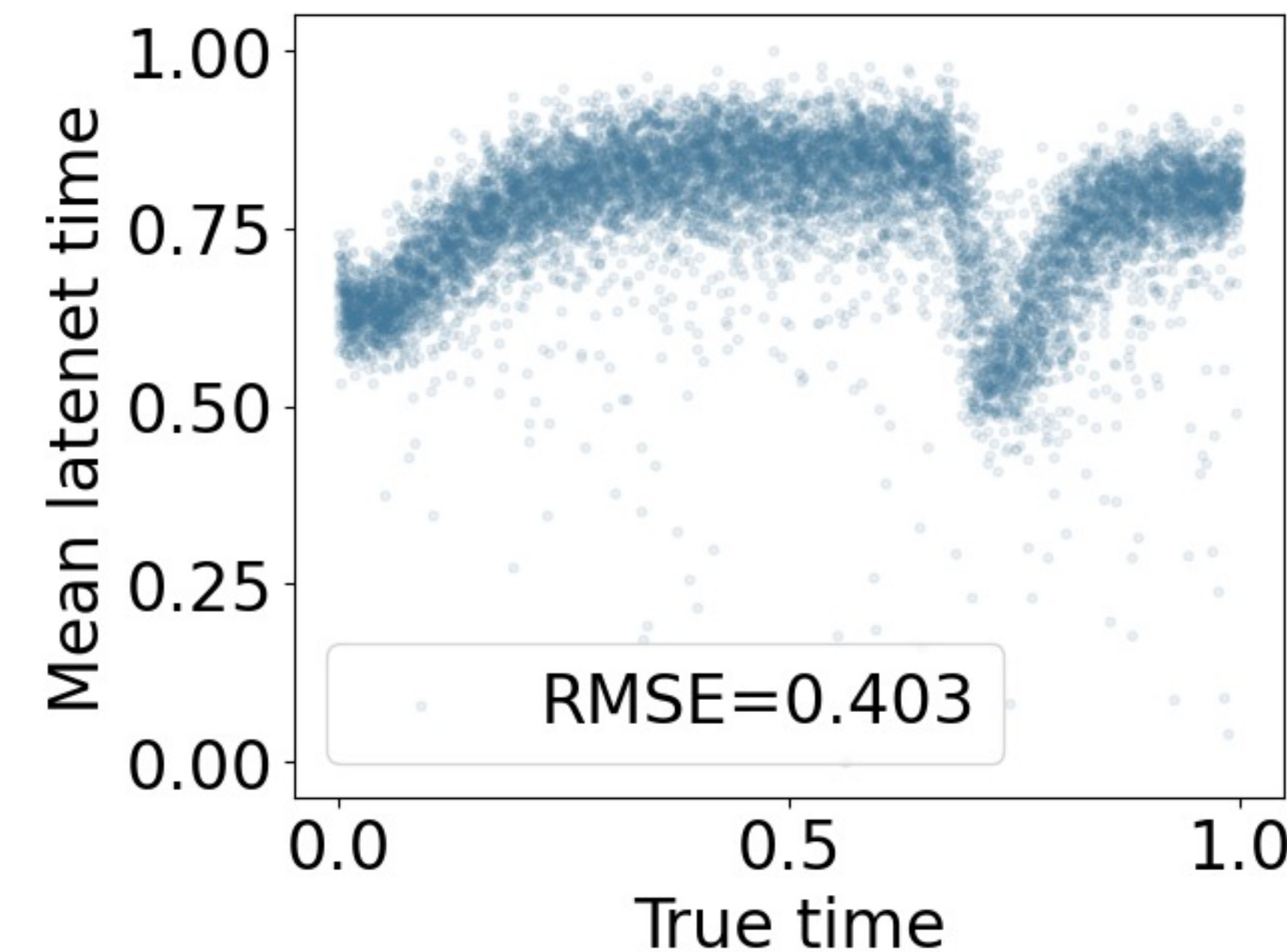

Supplement: S23 Fig — Monocle 3 [6], Slingshot [9], diffusion pseudotime [43] and veloVI [13] are applied on simulation data used in Fig 2. Inferred time is plotted against true time, where x axis is the true simulation time and y axis is corresponding inferred time normalized between 0 and 1. RMSE stands for root mean square error of inferred time. (PDF) [file pcbi.1012752.s024.pdf]

**a**

Monocle 3

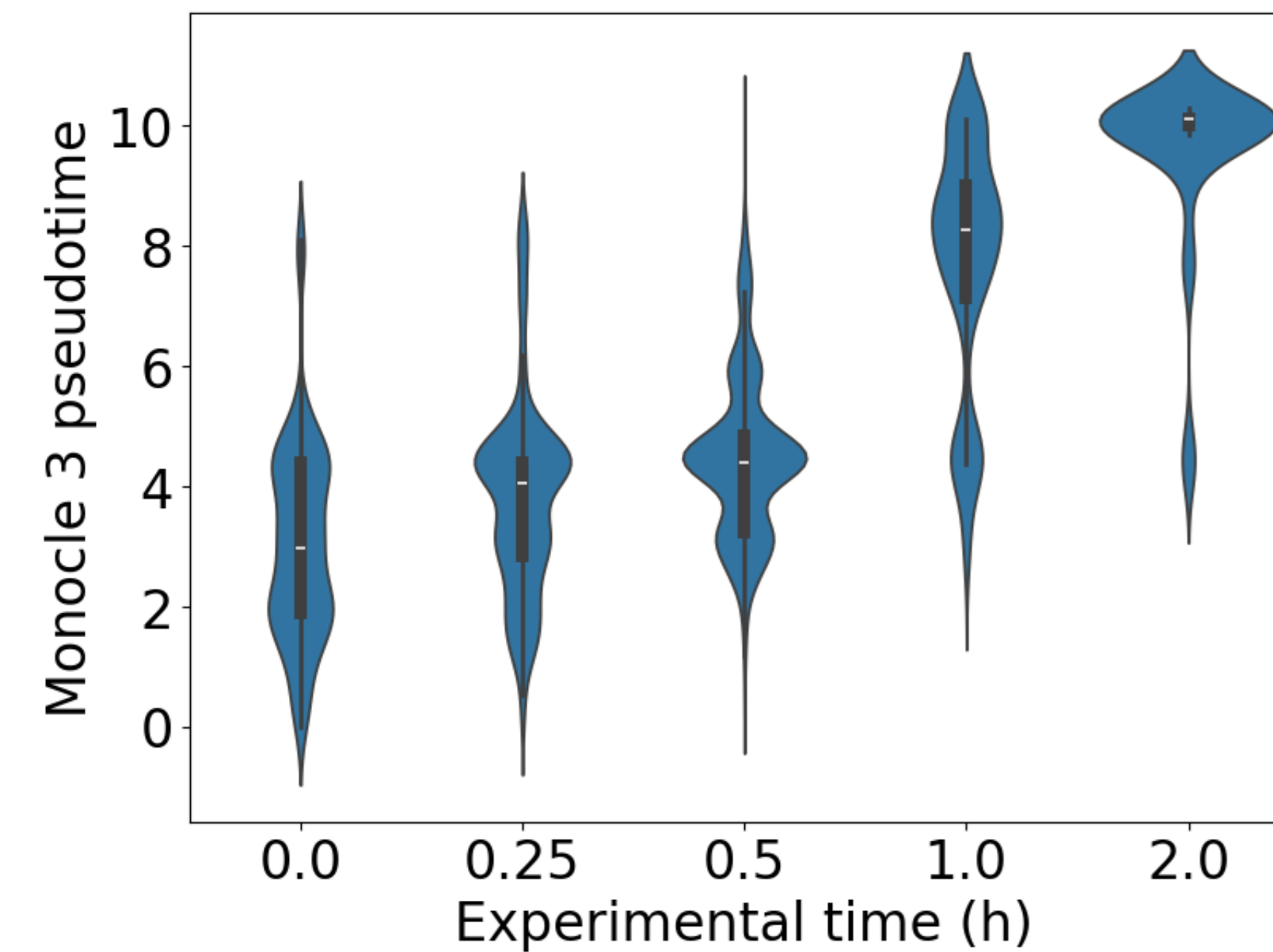**b**

Slingshot

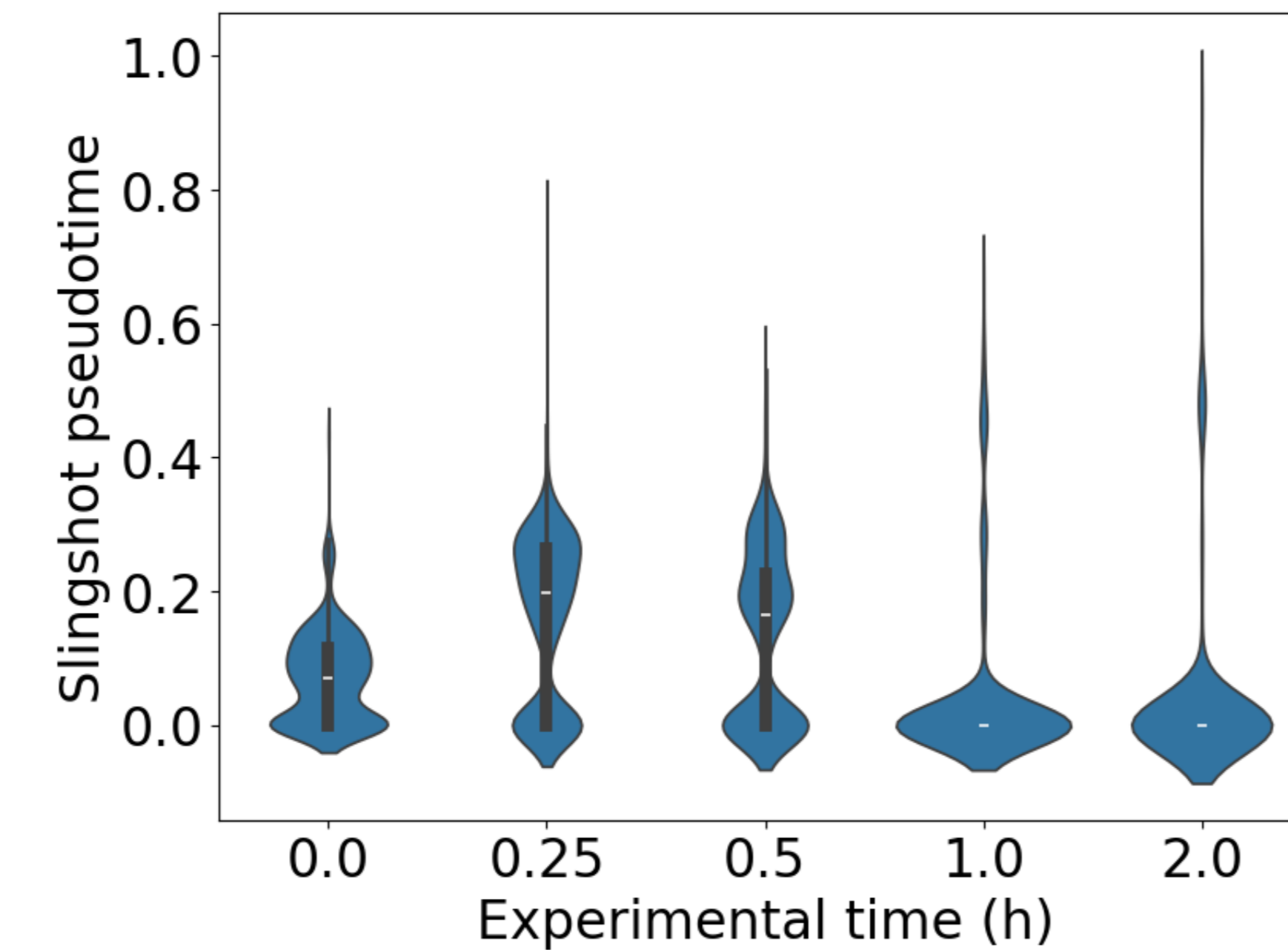**c**

Diffusion pseudotime

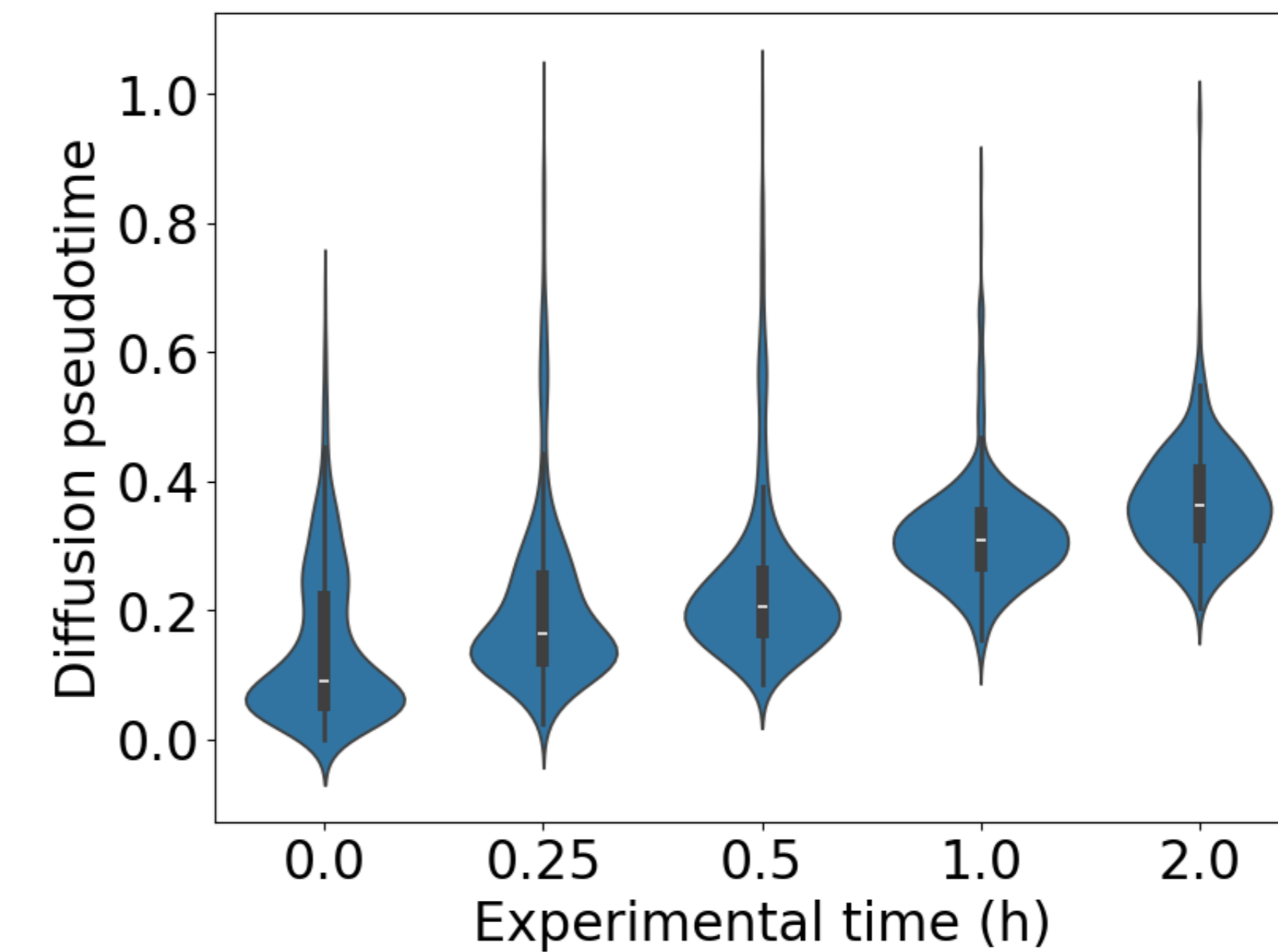**d**

veloVI

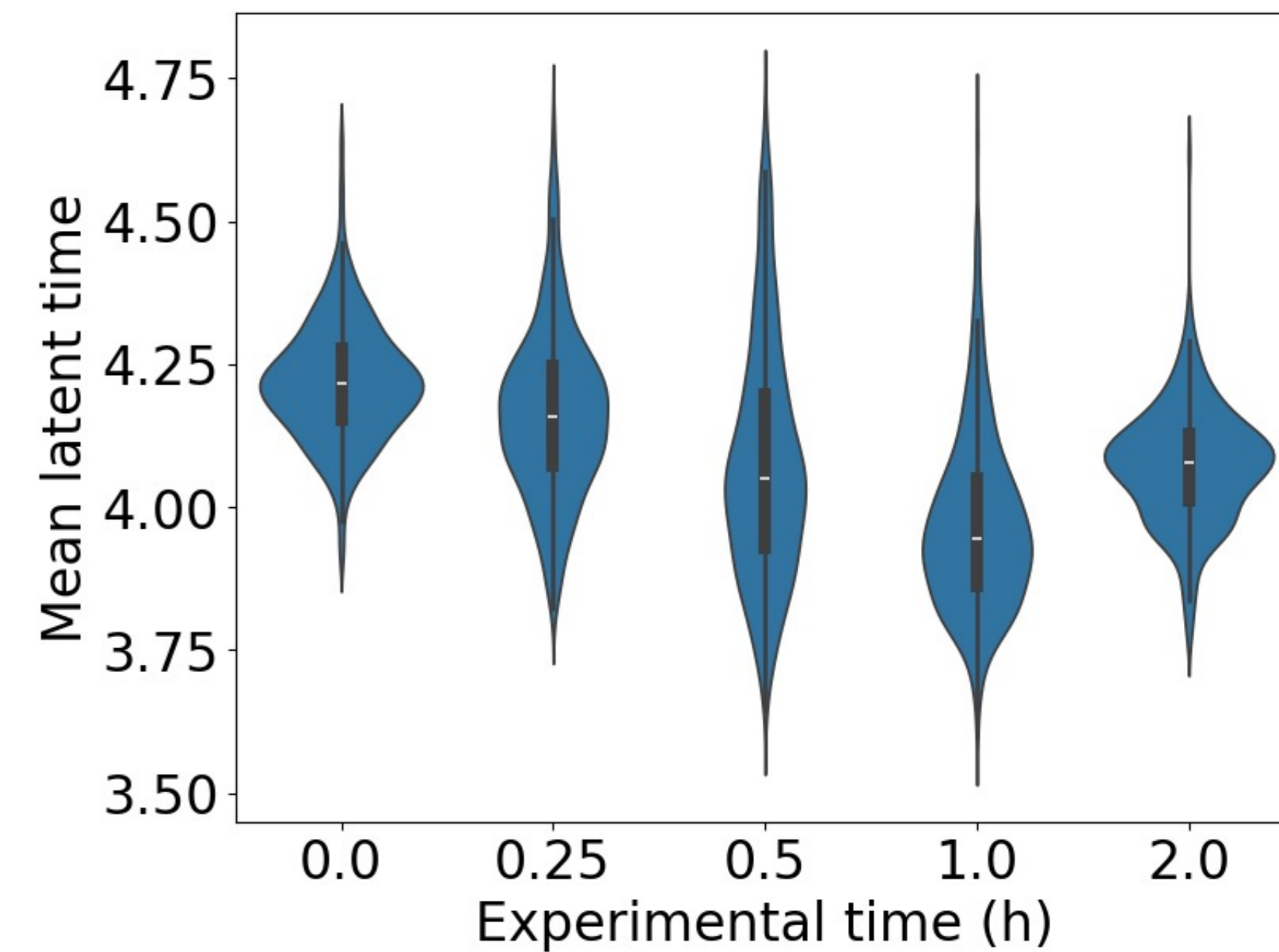

Supplement: S24 Fig — Monocle 3 [6], Slingshot [9], diffusion pseudotime [43] and veloVI [13] are applied on Neuron data used in S21 Fig to generate violin plots comparing inferred time to experimental time. In these plots, the x-axis represents the experimental time, while the y-axis shows the corresponding inferred time. (PDF) [file pcbi.1012752.s025.pdf]
