# Supplementary material for: Trajectory inference from single-cell genomics data with a process time model
Source: PLoS Comput Biol. 2025 Jan 21;21(1):e1012752. doi: 10.1371/journal.pcbi.1012752 (PMC11760028; doi:10.1371/journal.pcbi.1012752)
Supplement: S4 Fig — a) The same data from 4 Poisson mixtures as in S1 Fig. b) To compare the trajectory and Poisson mixture models, AIC scores of Poisson mixtures model and trajectory model are compared on 20 simulations with different random parameter sets. Dots below y=x indicate Poisson mixture model is better. (PDF) [file pcbi.1012752.s005.pdf]

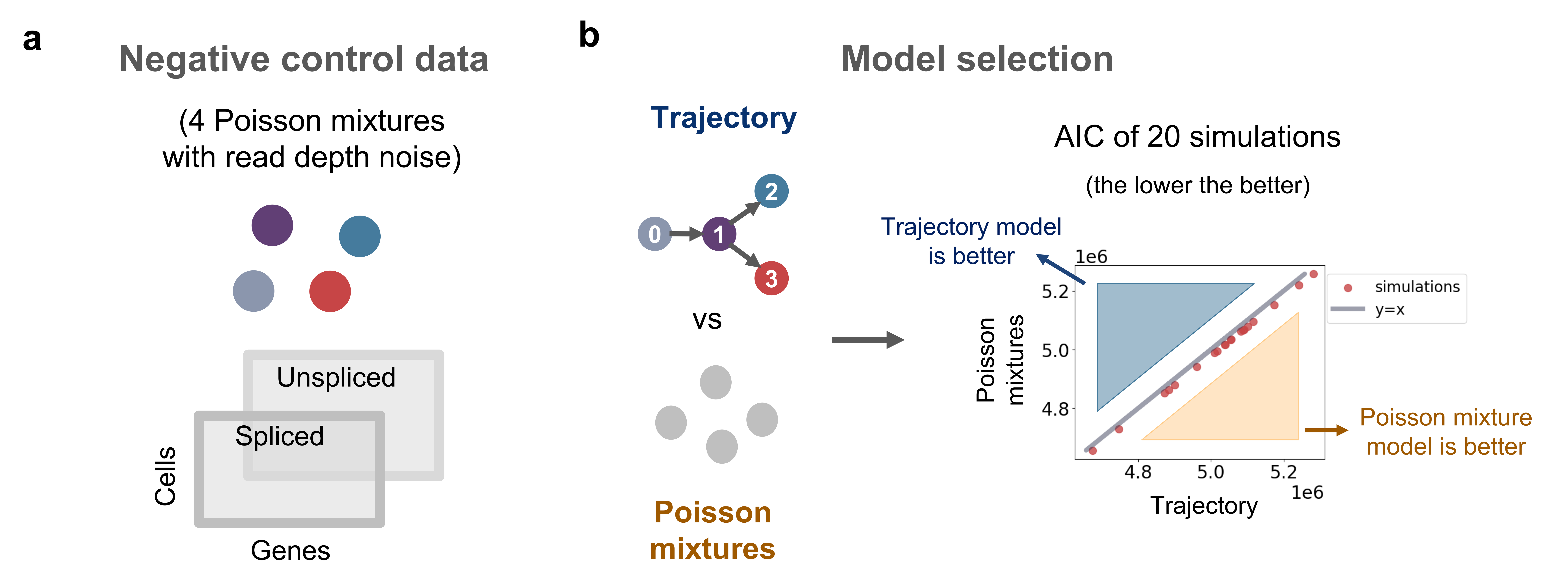
a
Negative control data
(4 Poisson mixtures
with read depth noise)
Unspliced
Spliced
Cells
Genes
b
Model selection
Trajectory
0
1
2
3
vs
Poisson
mixtures
AIC of 20 simulations
(the lower the better)
Trajectory model
is better
1e6
5.2
5.0
4.8
Poisson
mixtures
4.8
5.0
5.2
1e6
Trajectory
simulations
y=x
Poisson mixture
model is better
