# Supplementary material for: Trajectory inference from single-cell genomics data with a process time model
Source: PLoS Comput Biol. 2025 Jan 21;21(1):e1012752. doi: 10.1371/journal.pcbi.1012752 (PMC11760028; doi:10.1371/journal.pcbi.1012752)
Supplement: S6 Fig — a) Absolute errors with respect to the true values of parameters. b) Distribution of the smallest eigenvalues of the Fisher information matrix of each gene. c) Marginal likelihood (ELBO) and varied parameters compared to true parameters. The difference between varied parameters and true parameters are the eigenvectors corresponding to the smallest eigenvalues of the Fisher information matrix, divided by the square root of the respective eigenvalues, specific to variable genes. (PDF) [file pcbi.1012752.s007.pdf]

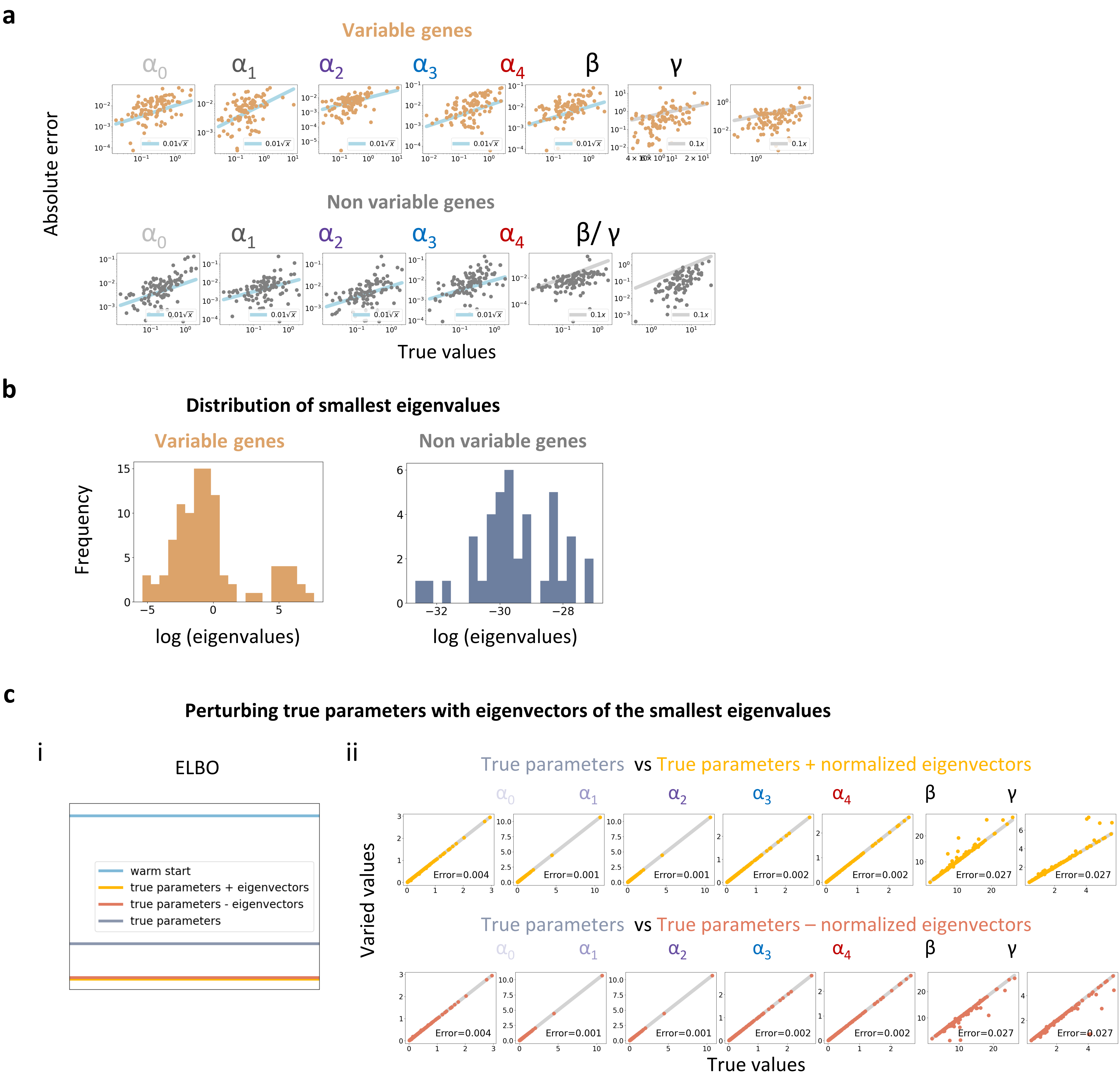
a
Variable genes
α0
α1
α2
α3
α4
β
γ
Absolute error
0.01√x
0.1x
Non variable genes
β/ γ
True values
b
Distribution of smallest eigenvalues
Variable genes
Non variable genes
Frequency
log (eigenvalues)
c
Perturbing true parameters with eigenvectors of the smallest eigenvalues
i
ELBO
warm start
true parameters + eigenvectors
true parameters - eigenvectors
true parameters
ii
True parameters vs True parameters + normalized eigenvectors
True parameters vs True parameters – normalized eigenvectors
Varied values
Error=0.004
Error=0.001
Error=0.002
Error=0.027
True values
