# Supplementary material for: Trajectory inference from single-cell genomics data with a process time model
Source: PLoS Comput Biol. 2025 Jan 21;21(1):e1012752. doi: 10.1371/journal.pcbi.1012752 (PMC11760028; doi:10.1371/journal.pcbi.1012752)
Supplement: S7 Fig — a) Estimation errors for datasets with varying cell numbers. The trajectory structures are the same as in S2a Fig. For time, error is root mean square error. For α , β , γ, error is mean normalized error as described in the Section Simulations. Estimation errors of different cell numbers. b) Running time of 100 epochs on a single core on datasets with varying cell numbers. (PDF) [file pcbi.1012752.s008.pdf]

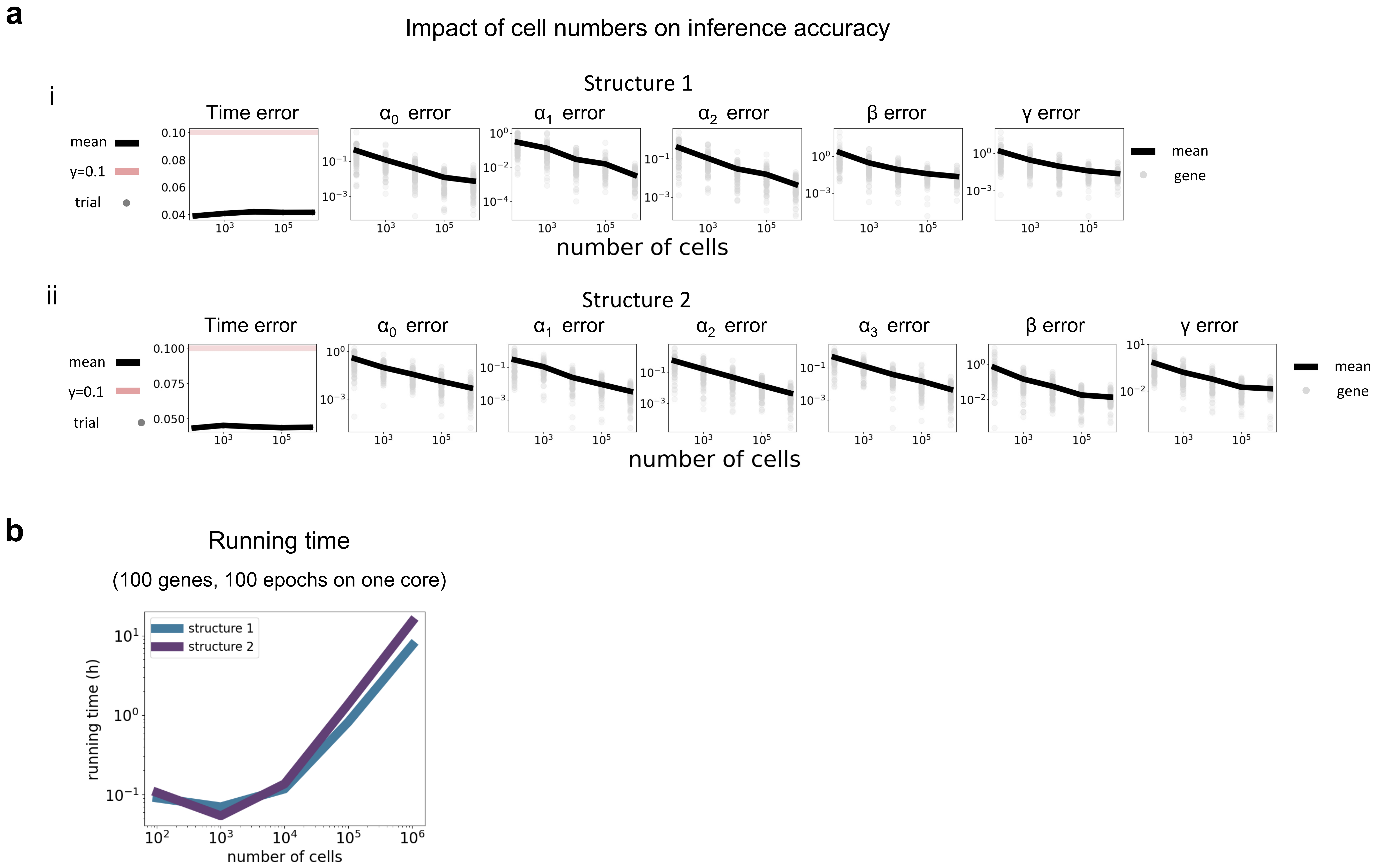

a
Impact of cell numbers on inference accuracy
i
Structure 1
Time error
α0 error
α1 error
α2 error
β error
γ error
mean
y=0.1
trial
mean
gene
number of cells
ii
Structure 2
Time error
α0 error
α1 error
α2 error
α3 error
β error
γ error
mean
y=0.1
trial
mean
gene
number of cells
b
Running time
(100 genes, 100 epochs on one core)
structure 1
structure 2
running time (h)
number of cells
