# Supplementary material for: Trajectory inference from single-cell genomics data with a process time model
Source: PLoS Comput Biol. 2025 Jan 21;21(1):e1012752. doi: 10.1371/journal.pcbi.1012752 (PMC11760028; doi:10.1371/journal.pcbi.1012752)
Supplement: S10 Fig — a) The trajectory structure used in this figure. b) Impact of counts means on inference accuracy. Datasets with increasing counts means are generated by increasing the mean parameters μ in log-normal distributions for α. c) Results on 10 random parameter sets with different distributions for α. d) Impact of cell numbers on inference accuracy on simulations with different distributions for α. (PDF) [file pcbi.1012752.s011.pdf]

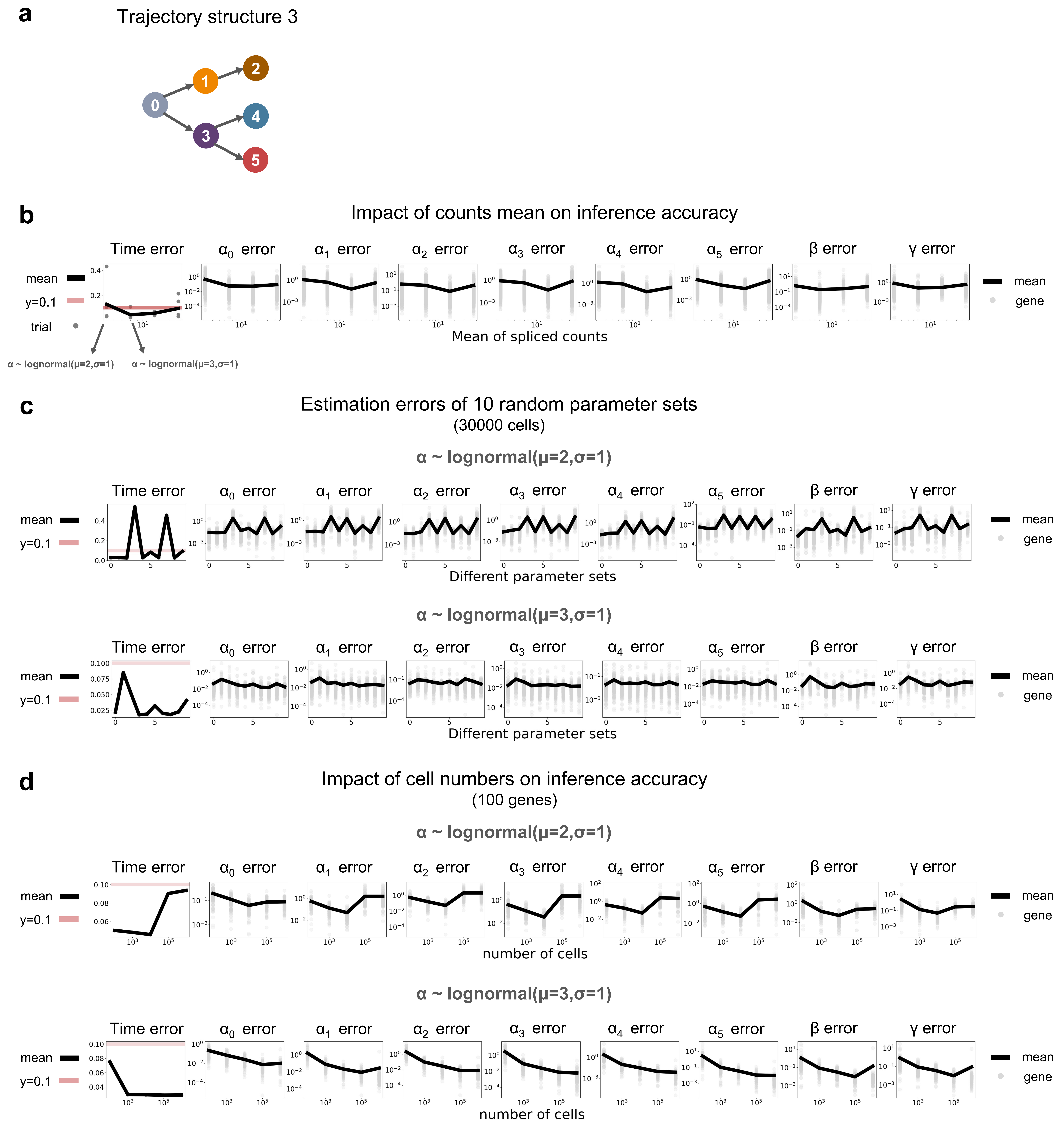
a
Trajectory structure 3
0
1
2
3
4
5
b
Impact of counts mean on inference accuracy
Time error
α0 error
α1 error
α2 error
α3 error
α4 error
α5 error
β error
γ error
mean
y=0.1
trial
gene
Mean of spliced counts
α ~ lognormal(μ=2,σ=1)
α ~ lognormal(μ=3,σ=1)
c
Estimation errors of 10 random parameter sets
(30000 cells)
α ~ lognormal(μ=2,σ=1)
Different parameter sets
α ~ lognormal(μ=3,σ=1)
Different parameter sets
d
Impact of cell numbers on inference accuracy
(100 genes)
α ~ lognormal(μ=2,σ=1)
number of cells
α ~ lognormal(μ=3,σ=1)
number of cells
