# Supplementary material for: Trajectory inference from single-cell genomics data with a process time model
Source: PLoS Comput Biol. 2025 Jan 21;21(1):e1012752. doi: 10.1371/journal.pcbi.1012752 (PMC11760028; doi:10.1371/journal.pcbi.1012752)
Supplement: S12 Fig — The trajectory structures are the same as in S2a Fig. For time, error is root mean square error. For α , β , γ, error is mean normalized error as described in the Section Simulations. a) Estimation errors as read depth noise increases. b) Estimation errors as gene-wise Gamma noise increases. c) Impact of gene-wise Gamma noise on model selection on clusters data. Same as in S4 Fig except Gamma noise with CV2 = 1 was added to Poisson mixtures to generate simulation data (Section Simulations). d) Impact of gene-wise Gamma noise on model selection of trajectory structure. Same as in Fig 2j except Gamma noise with CV2 = 1 was added in simulation. (PDF) [file pcbi.1012752.s013.pdf]

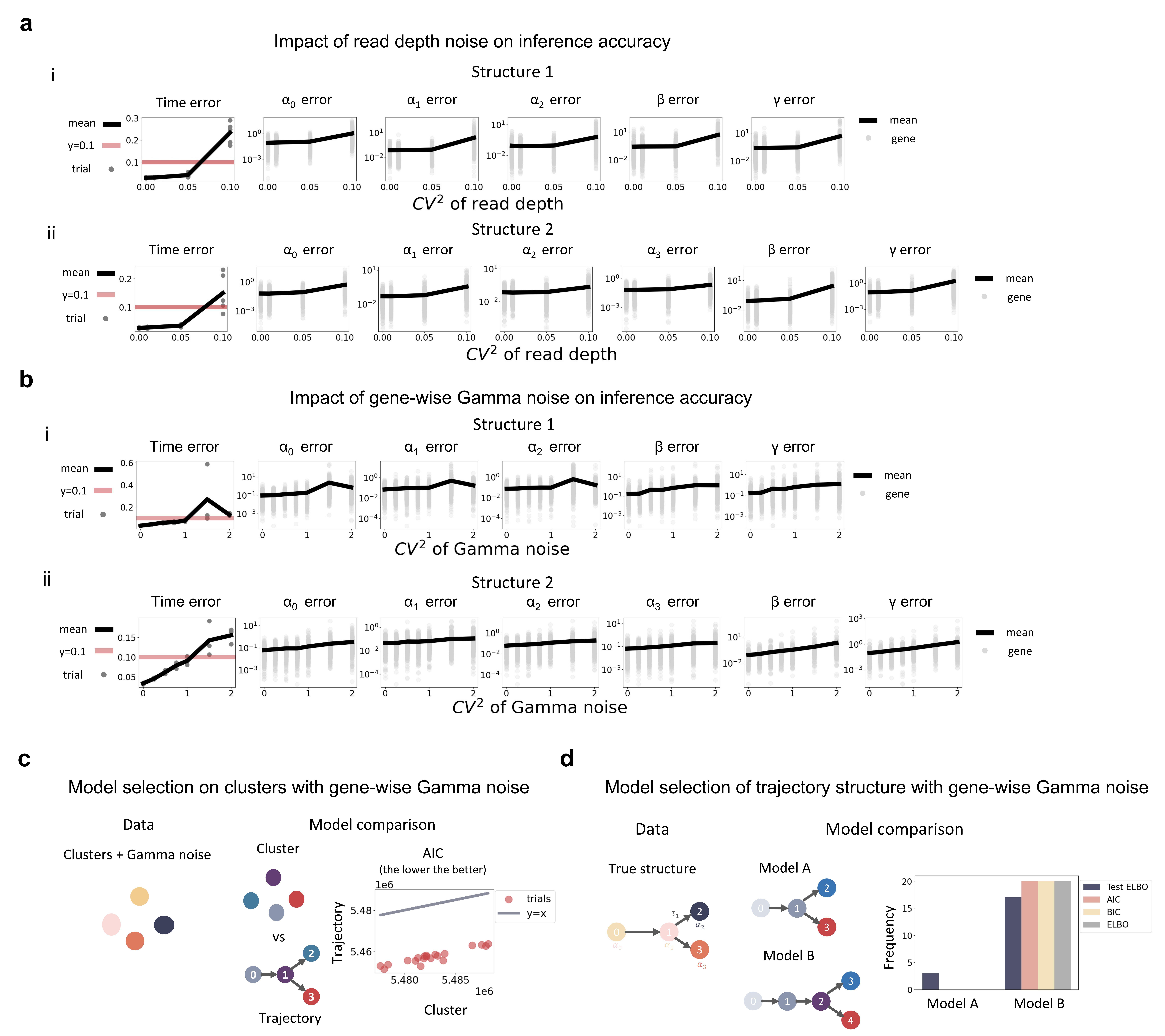
a
Impact of read depth noise on inference accuracy
i
Structure 1
Time error
α_0 error
α_1 error
α_2 error
β error
γ error
mean
y=0.1
trial
gene
CV^2 of read depth
ii
Structure 2
Time error
α_0 error
α_1 error
α_2 error
α_3 error
β error
γ error
mean
y=0.1
trial
gene
CV^2 of read depth
b
Impact of gene-wise Gamma noise on inference accuracy
i
Structure 1
Time error
α_0 error
α_1 error
α_2 error
β error
γ error
mean
y=0.1
trial
gene
CV^2 of Gamma noise
ii
Structure 2
Time error
α_0 error
α_1 error
α_2 error
α_3 error
β error
γ error
mean
y=0.1
trial
gene
CV^2 of Gamma noise
c
Model selection on clusters with gene-wise Gamma noise
Data
Clusters + Gamma noise
Model comparison
Cluster
vs
Trajectory
AIC
(the lower the better)
1e6
trials
y=x
Trajectory
Cluster
d
Model selection of trajectory structure with gene-wise Gamma noise
Data
True structure
Model comparison
Model A
Model B
Test ELBO
AIC
BIC
ELBO
Frequency
Model A
Model B
