# Supplementary material for: Trajectory inference from single-cell genomics data with a process time model
Source: PLoS Comput Biol. 2025 Jan 21;21(1):e1012752. doi: 10.1371/journal.pcbi.1012752 (PMC11760028; doi:10.1371/journal.pcbi.1012752)
Supplement: S13 Fig — The trajectory structures are the same as in S2a Fig. For time, error is root mean square error. For α , β , γ, error is mean normalized error as described in the Section Simulations. a) i Schematics of phase plots with increasing timescale. ii Estimation errors as time scale increases. b) i Schematics of phase plots with the ratio of γ to β increasing while keeping their product constant. ii Estimation errors as γβ increases. (PDF) [file pcbi.1012752.s014.pdf]

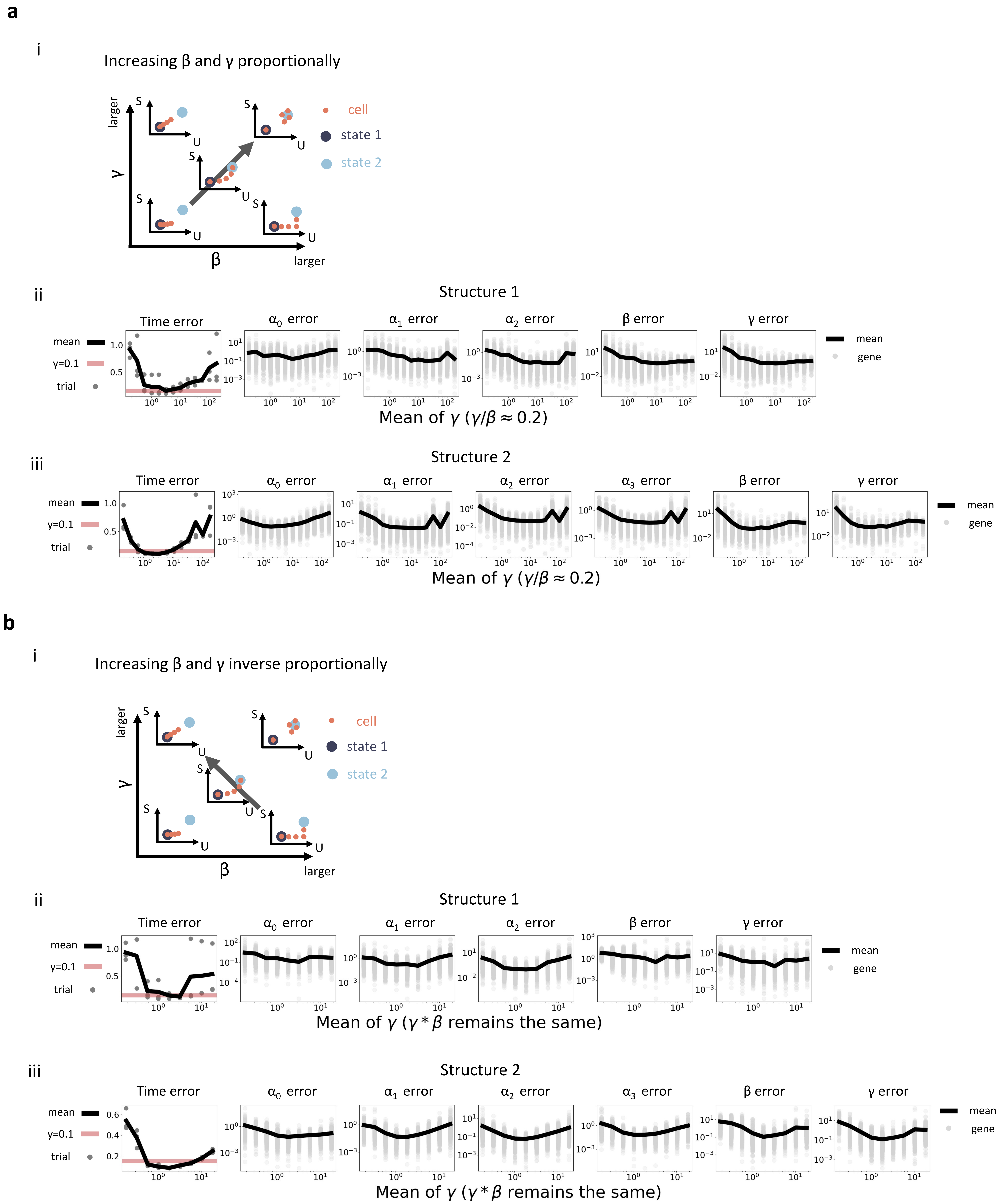
a
i
Increasing β and γ proportionally
larger
γ
β
larger
S
U
cell
state 1
state 2
ii
Structure 1
Time error
mean
γ=0.1
trial
α_0 error
α_1 error
α_2 error
β error
γ error
mean
gene
Mean of γ ($\gamma/\beta \approx 0.2$)
iii
Structure 2
Time error
mean
γ=0.1
trial
α_0 error
α_1 error
α_2 error
α_3 error
β error
γ error
mean
gene
Mean of γ ($\gamma/\beta \approx 0.2$)
b
i
Increasing β and γ inverse proportionally
larger
γ
β
larger
S
U
cell
state 1
state 2
ii
Structure 1
Time error
mean
γ=0.1
trial
α_0 error
α_1 error
α_2 error
β error
γ error
mean
gene
Mean of γ ($\gamma * \beta$ remains the same)
iii
Structure 2
Time error
mean
γ=0.1
trial
α_0 error
α_1 error
α_2 error
α_3 error
β error
γ error
mean
gene
Mean of γ ($\gamma * \beta$ remains the same)
