# Supplementary material for: Trajectory inference from single-cell genomics data with a process time model
Source: PLoS Comput Biol. 2025 Jan 21;21(1):e1012752. doi: 10.1371/journal.pcbi.1012752 (PMC11760028; doi:10.1371/journal.pcbi.1012752)
Supplement: S14 Fig — The trajectory structures are the same as in S2a Fig. For time, error is root mean square error. For α , β , γ, error is mean normalized error as described in the Section Simulations. a) Schematics of sampling distributions used in simulation with decreasing uniformity. The sampling distributions were gradually changing from uniform distribution to Gaussian distribution. Right plot shows the entropy of the sampling distributions. b) Estimation errors as uniformity decreases under uniform prior. c) Estimation errors as uniformity decreases warm started with correct position under true prior. Fitting was initialized with posteriors calculated under true parameters, and empirical distribution of process time of samples were provided as prior for the sampling distribution. (PDF) [file pcbi.1012752.s015.pdf]

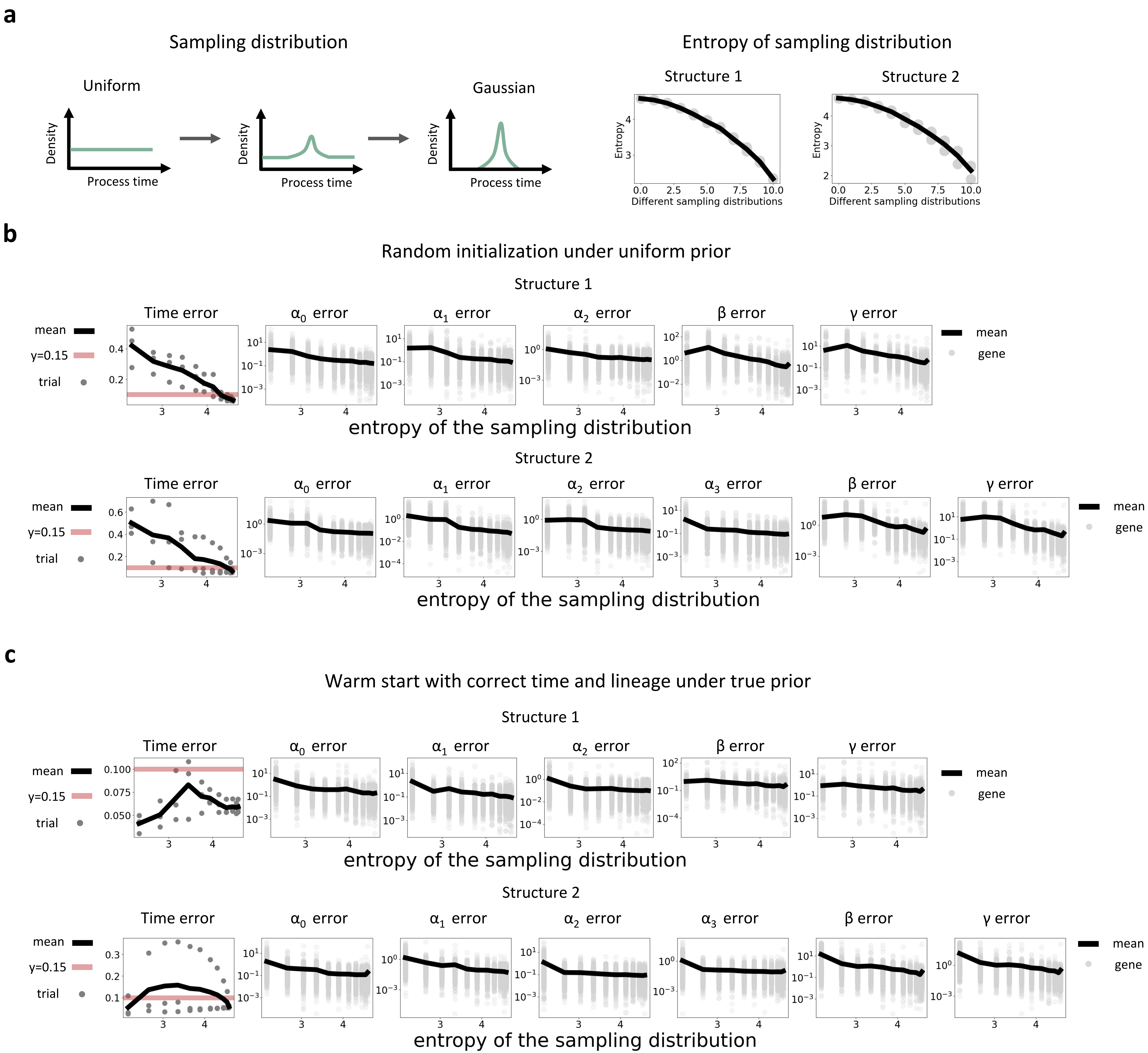
a
Sampling distribution
Uniform
Gaussian
Density
Process time
Entropy of sampling distribution
Structure 1
Structure 2
Entropy
Different sampling distributions
b
Random initialization under uniform prior
Structure 1
Time error
α0 error
α1 error
α2 error
β error
γ error
mean
y=0.15
trial
gene
entropy of the sampling distribution
Structure 2
Time error
α0 error
α1 error
α2 error
α3 error
β error
γ error
mean
y=0.15
trial
gene
entropy of the sampling distribution
c
Warm start with correct time and lineage under true prior
Structure 1
Time error
α0 error
α1 error
α2 error
β error
γ error
mean
y=0.15
trial
gene
entropy of the sampling distribution
Structure 2
Time error
α0 error
α1 error
α2 error
α3 error
β error
γ error
mean
y=0.15
trial
gene
entropy of the sampling distribution
