# Supplementary material for: Trajectory inference from single-cell genomics data with a process time model
Source: PLoS Comput Biol. 2025 Jan 21;21(1):e1012752. doi: 10.1371/journal.pcbi.1012752 (PMC11760028; doi:10.1371/journal.pcbi.1012752)
Supplement: S15 Fig — a) Schematics of three probable failure factors: clusters data, fast timescale, concentrated sampling distribution. Two example simulations were used for each case and the gray arrows indicate the their difference. i The two simulations are the cluster data in S4 Fig and noisy cluster data in S12c Fig respectively. ii The two simulations are the 5th and 13th instances of structure 1 in S13b Fig. iii The two simulations are the 1st and 11th instances of structure 2 in S14b Fig. b) AIC vs. correlation of mean process time of 100 random initializations in different scenarios. Results of two example simulations were showed. The x axis is the correlation of mean process time between each initialization and the best one. The gray arrows correspond to those in a. c) AIC vs correlation of mean process time of 20 bootstrap samples in different scenarios. Results of the two example simulations were presented in the same position as in b. The x axis is the correlation of mean process time between each bootstrap sample and the original one (which is the best one in b). The gray arrows correspond to those in a. (PDF) [file pcbi.1012752.s016.pdf]

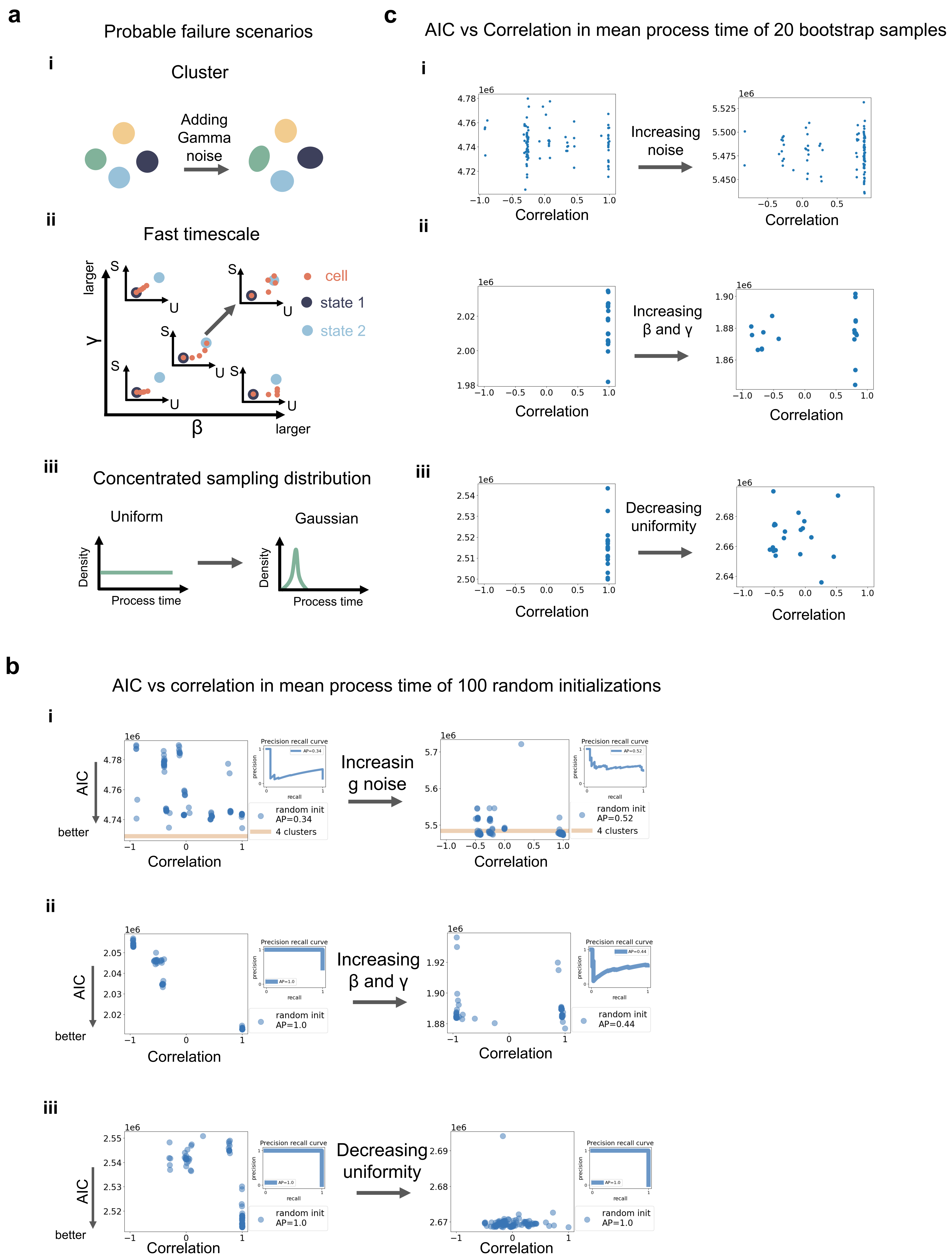

a
Probable failure scenarios
i
Cluster
Adding Gamma noise
ii
Fast timescale
cell
state 1
state 2
S
U
larger
γ
β
larger
iii
Concentrated sampling distribution
Uniform
Gaussian
Density
Process time
b
AIC vs correlation in mean process time of 100 random initializations
i
AIC
better
Correlation
Precision recall curve
random init AP=0.34
4 clusters
Increasin g noise
random init AP=0.52
4 clusters
ii
Increasing β and γ
random init AP=1.0
random init AP=0.44
iii
Decreasing uniformity
random init AP=1.0
random init AP=1.0
c
AIC vs Correlation in mean process time of 20 bootstrap samples
i
Increasing noise
Correlation
ii
Increasing β and γ
iii
Decreasing uniformity
