# Supplementary material for: Trajectory inference from single-cell genomics data with a process time model
Source: PLoS Comput Biol. 2025 Jan 21;21(1):e1012752. doi: 10.1371/journal.pcbi.1012752 (PMC11760028; doi:10.1371/journal.pcbi.1012752)
Supplement: S18 Fig — a) Schematics of T cells from PBMC dataset and PCA plots. b) The fit trajectory structure and AIC scores of 100 random initializations (blue dots) compared to those of 3 clusters (Poisson mixtures) model (yellow line). AP stands for average precision. (PDF) [file pcbi.1012752.s019.pdf]

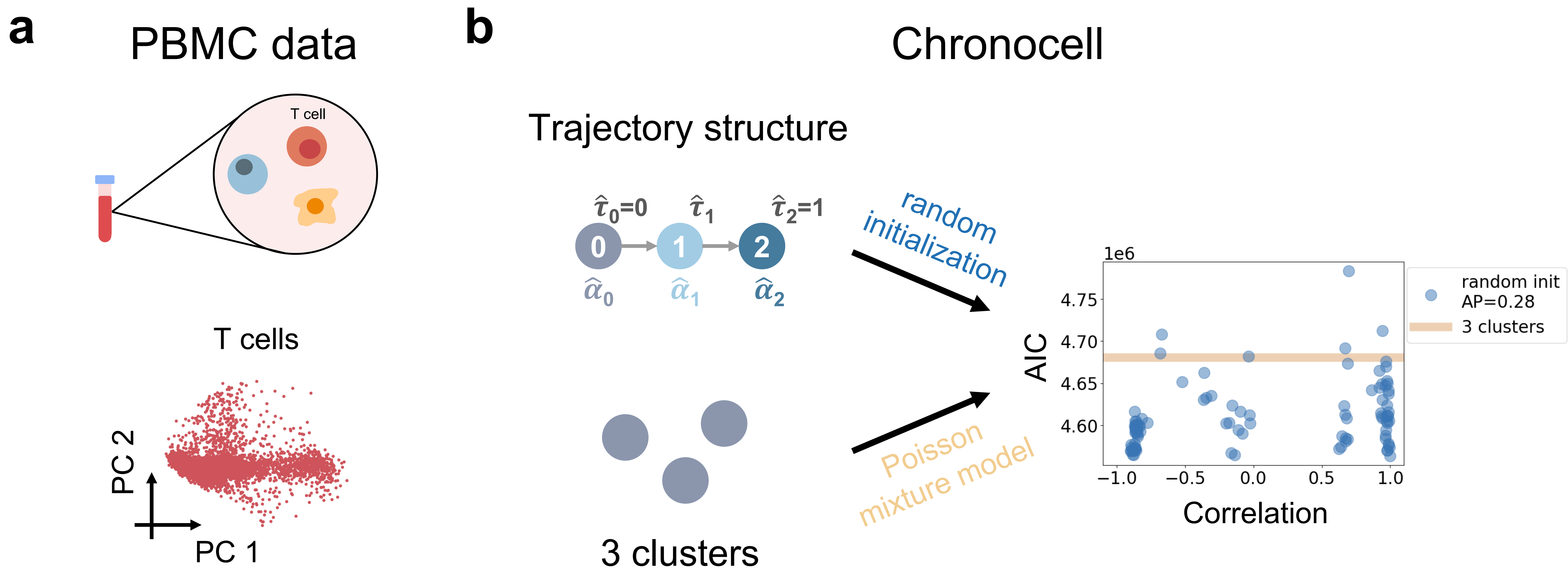

a
PBMC data
T cell
T cells
PC 2
PC 1
b
Chronocell
Trajectory structure
$\hat{\tau}_0$=0
$\hat{\tau}_1$
$\hat{\tau}_2$=1
0
1
2
$\hat{\alpha}_0$
$\hat{\alpha}_1$
$\hat{\alpha}_2$
random initialization
3 clusters
Poisson mixture model
1e6
4.75
4.70
4.65
4.60
AIC
−1.0
−0.5
0.0
0.5
1.0
Correlation
random init AP=0.28
3 clusters
