# Supplementary material for: Trajectory inference from single-cell genomics data with a process time model
Source: PLoS Comput Biol. 2025 Jan 21;21(1):e1012752. doi: 10.1371/journal.pcbi.1012752 (PMC11760028; doi:10.1371/journal.pcbi.1012752)
Supplement: S19 Fig — a) AIC scores and mean process time correlations of 100 random initializations (blue dots) compared to those of warm start (red line) as well as 3 clusters (Poisson mixtures) model (yellow line). AP stands for average precision. Mean process time of the initialization with lowest AIC is indicated in blue on the same PCA plot as in a. b) AIC scores and mean process time correlations of 100 bootstrap samples. The x axis is the Pearson’s correlation between the mean process time of each bootstrap and the those of original data, i.e., the plotted one in a. c) Distribution of remaining squared coefficient of variance of 49 genes used in the fitting. Remaining squared coefficient of variance is calculated by dividing the remaining unexplained variance by mean squared. (PDF) [file pcbi.1012752.s020.pdf]

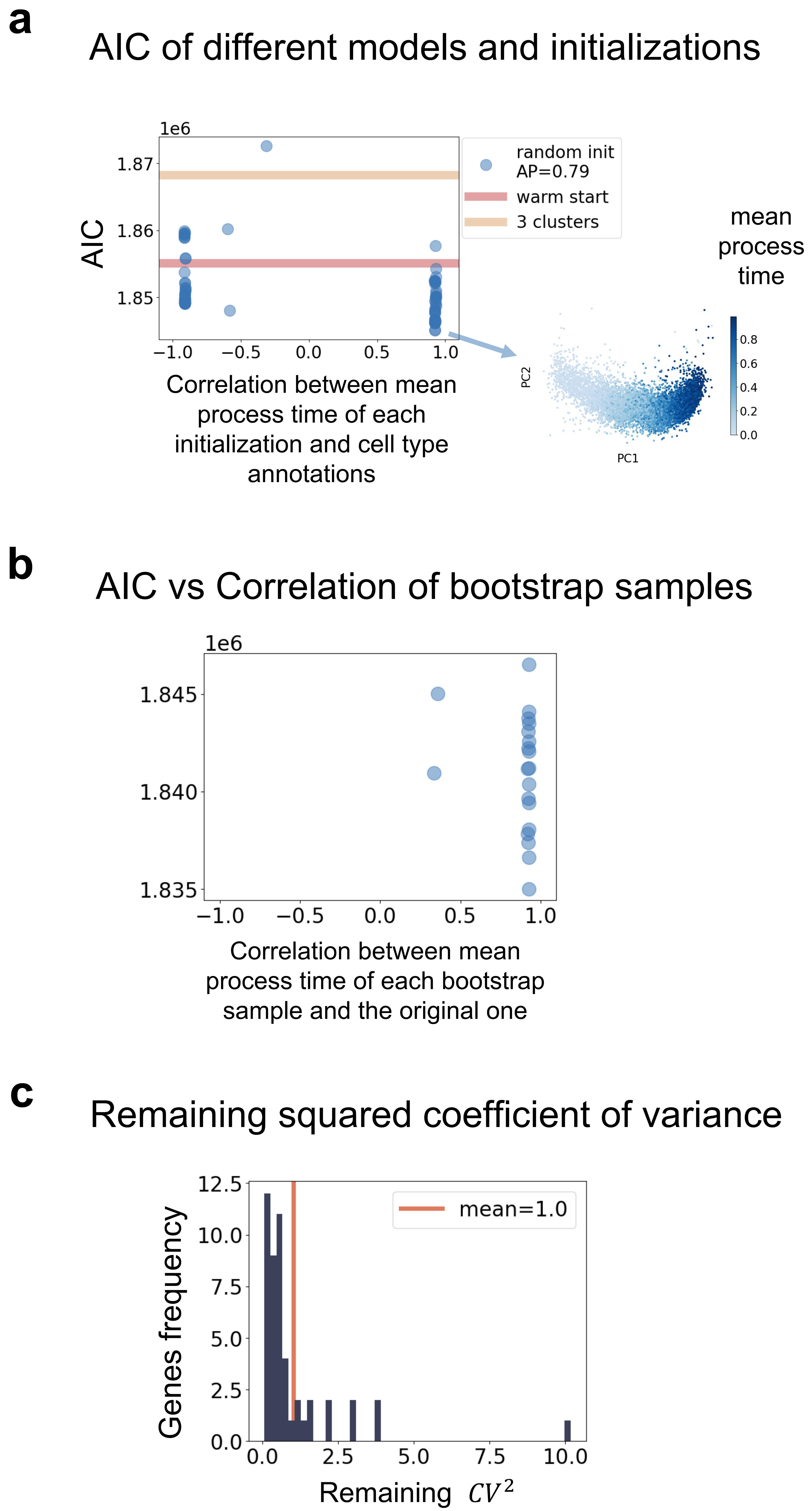
a
AIC of different models and initializations
1e6
1.87
1.86
1.85
AIC
−1.0
−0.5
0.0
0.5
1.0
Correlation between mean process time of each initialization and cell type annotations
random init AP=0.79
warm start
3 clusters
mean process time
PC2
PC1
0.8
0.6
0.4
0.2
0.0
b
AIC vs Correlation of bootstrap samples
1e6
1.845
1.840
1.835
−1.0
−0.5
0.0
0.5
1.0
Correlation between mean process time of each bootstrap sample and the original one
c
Remaining squared coefficient of variance
12.5
10.0
7.5
5.0
2.5
0.0
Genes frequency
mean=1.0
0.0
2.5
5.0
7.5
10.0
Remaining CV^2
