# Supplementary material for: Trajectory inference from single-cell genomics data with a process time model
Source: PLoS Comput Biol. 2025 Jan 21;21(1):e1012752. doi: 10.1371/journal.pcbi.1012752 (PMC11760028; doi:10.1371/journal.pcbi.1012752)
Supplement: S21 Fig — Fitting was warm started from delta distribution at physical time under different sampling distribution priors using the shown trajectory structure. a) The assumed sampling distribution. For iii, uniform distribution is assumed for cells from all time points. b) The fit trajectory structure and inferred mean process time indicated in blue on the PCA plot. c) Violin plots of mean process time of cells with different labeling times. Three blue bars represent the mean and extremes. (PDF) [file pcbi.1012752.s022.pdf]

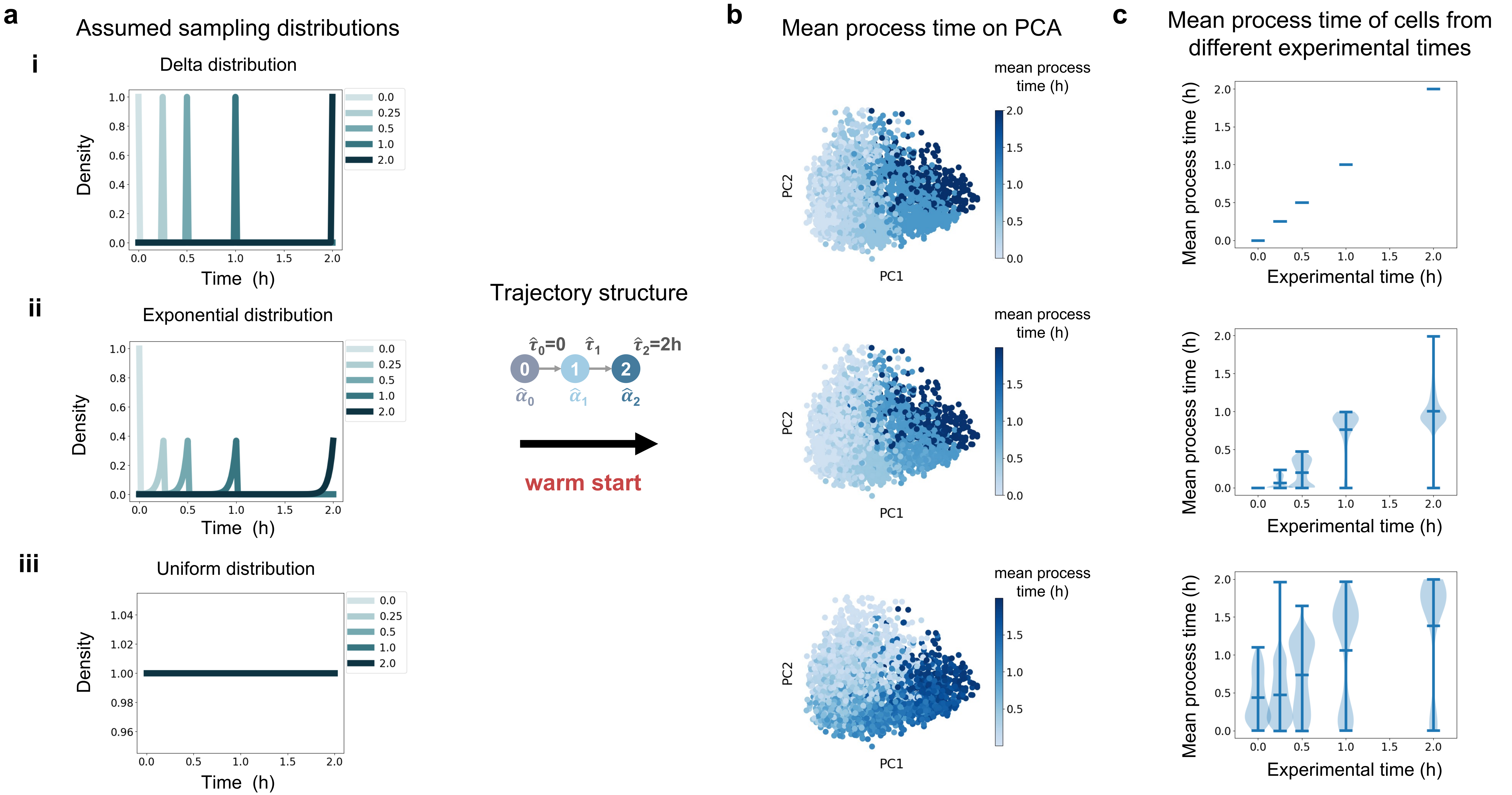

a
Assumed sampling distributions
i
Delta distribution
ii
Exponential distribution
iii
Uniform distribution
Density
Time (h)
0.0
0.25
0.5
1.0
2.0
Trajectory structure
$\hat{\tau}_0$=0
$\hat{\tau}_1$
$\hat{\tau}_2$=2h
$\hat{\alpha}_0$
$\hat{\alpha}_1$
$\hat{\alpha}_2$
warm start
b
Mean process time on PCA
mean process time (h)
PC1
PC2
c
Mean process time of cells from different experimental times
Mean process time (h)
Experimental time (h)
