# Supplementary material for: Trajectory inference from single-cell genomics data with a process time model
Source: PLoS Comput Biol. 2025 Jan 21;21(1):e1012752. doi: 10.1371/journal.pcbi.1012752 (PMC11760028; doi:10.1371/journal.pcbi.1012752)
Supplement: S22 Fig — Chronocell, Monocle 3 [6], Slingshot [9], diffusion pseudotime [43] and veloVI [13] are applied on simulation generated using dyngen [24]. Inferred time is plotted against true time, where x axis is the true simulation time normalized between 0 and 1 and y axis is corresponding inferred time normalized between 0 and 1. RMSE stands for root mean square error of inferred time. a) The dyngen simulation projected into the first two principal component spaces. A bifurcation backbone is used. b) The fit trajectory structure and results of Chronocell. c) The results of Monocle 3. d) The results of Slingshot. e) The results of diffusion pseudotime. f) The results of veloVI. (PDF) [file pcbi.1012752.s023.pdf]

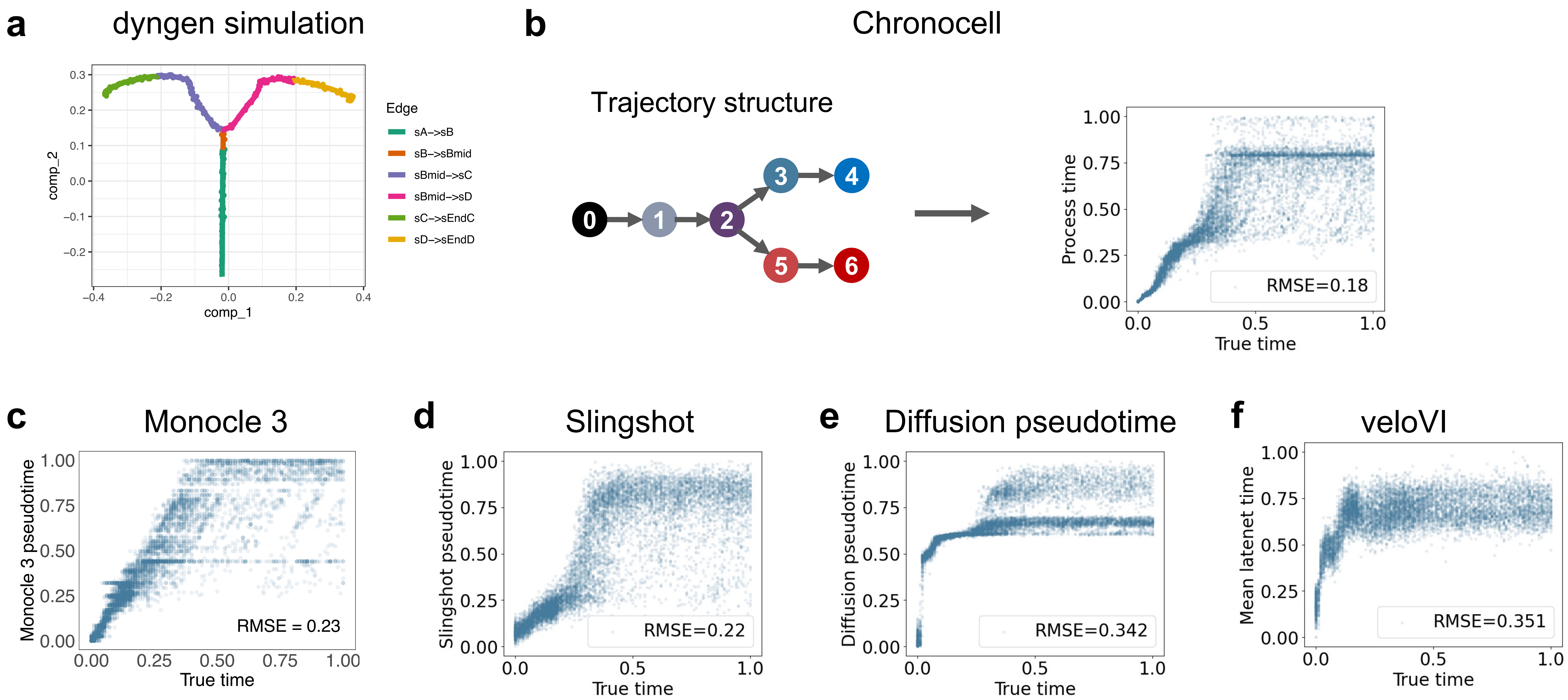
a
dyngen simulation
Edge
sA->sB
sB->sBmid
sBmid->sC
sBmid->sD
sC->sEndC
sD->sEndD
comp_1
comp_2
b
Chronocell
Trajectory structure
0
1
2
3
4
5
6
Process time
True time
RMSE=0.18
c
Monocle 3
Monocle 3 pseudotime
True time
RMSE = 0.23
d
Slingshot
Slingshot pseudotime
True time
RMSE=0.22
e
Diffusion pseudotime
Diffusion pseudotime
True time
RMSE=0.342
f
veloVI
Mean latenet time
True time
RMSE=0.351
