# Supplementary material for: Trajectory inference from single-cell genomics data with a process time model
Source: PLoS Comput Biol. 2025 Jan 21;21(1):e1012752. doi: 10.1371/journal.pcbi.1012752 (PMC11760028; doi:10.1371/journal.pcbi.1012752)
Supplement: Attachment — Submitted filename: Response to reviewers.pdf [file pcbi.1012752.s026.pdf]

Response to reviewers

Reviewer #1:
This manuscript presents the Chronocell method for single-cell RNA-seq data trajectory inference and proposes a "process time" concept, which provides more physical meaning compared to the widely acknowledged "pseudotime" concept. To enhance the manuscript's clarity and impact, I recommend addressing the following points:

(1) Clarify the number of topologies that the proposed method can infer.

We assume that the topologies of the trajectories are provided by the user and that the lineages can be represented as paths in a directed graph. Theoretically, there is no limit on the number of topologies that Chronocell can infer. However, in practice, many factors such as noise and the dynamical information in the data can affect inference accuracy, as discussed in Section 2.3. Following the suggestion in Question 3, we have added examples with more complex trajectories in Figure S10 and S5, and expanded Section 2.3 to explain how topology complexity affects inference, along with influencing factors like the mean counts. In summary, inference becomes more challenging as trajectory topologies increase in complexity.

(2) Provide a detailed, step-by-step explanation of the process from the gene expression matrix to the derivation of trajectory and process time.

We appreciate this suggestion; we are aiming for Chronocell to be useful and easily run and generalized by users. Therefore, we have revised Figure 1 to clarify the procedure from input to results, with the gene expression matrix being one of the inputs. It now clarifies that the process from the gene expression matrix to the derivation of trajectory and process time involves fitting a specified trajectory model on data. We have also added an explanation of the Chronocell pipeline in Section 4.2.1 for clarity.

(3) Test the method on more complex trajectories and with a larger number of cells. The trajectory illustrated in Figure 2, which only shows one bifurcation, is relatively simple.

We have tested two more complex trajectory structures: 1) three lineages with two bifurcations with 30,000 cells (Figure 10); 2) a disconnected cluster alongside a bifurcation with 15,000 cells (Figure S5). For the first trajectory structure, we evaluated inference accuracy on simulations with 30,000 cells using various random parameter sets, with the results included in Figure S10. We have also increased the cell number to nearly one million and found that it improves accuracy only if the mean counts are sufficiently high. We have added text to Section 2.3 explaining the impact of the complexity of topologies on inference and the requirement for high mean counts. The second structure is conceptually more complex, as it encompasses a subset of cells from a continuous trajectory and another subset from discrete clusters. We evaluated the inference results and demonstrated that model selection correctly identifies the appropriate model on disconnected cluster data by comparing it to both a cluster model and a continuous trajectory model in Figure S5.

(4) Evaluate the computational efficiency of the iterative procedure for datasets at the million-cell level. Assess the running time as the number of cells increases significantly, given the advancements in single-cell sequencing technology.

We have assessed the running time from 100 cells to one million cells and included the results in Figure S7 and S10. When the number of cells increases significantly above 10,000, the running time scales linearly to the number of cells.

(5) Include a comprehensive comparison of the proposed method with recent state-of-the-art methods to further demonstrate its effectiveness.

We have compared Chronocell with Slingshot, Monocle 3, diffusion pseudotime (scanpy) and recently published veloVI which also integrates trajectory inference with RNA velocity on simulated data in Figure 2, as well as simulations generated by dyngen. The results are presented in Figure S22 and S23 and mentioned in the discussion section. We have found that other methods can not correctly recover true time on data generated under the Chronocell model and have comparable or lower accuracy on dyngen-generated data.

(6) Expand the discussion comparing "process time" and "pseudotime," which is currently limited to Figure 2. While both trajectories for Slingshot and the proposed method appear correct, a more detailed comparison is essential to highlight the advantages of the proposed "process time."

We have expanded the discussion on 'process time' and 'pseudotime' in the Introduction. Figure 2 was designed to illustrate “pseudotime” can 'discover' superficially plausible dynamics on false positive cluster data. We revised Figure 2 and separated into Figure S1 and S4 to further clarify this distinction. Figure S1 demonstrates that descriptive methods can lead to false positives and lack a principled approach to address them. In contrast, Figure S4 shows that, with a model-based approach like Chronocell, we can apply classical model selection methods to compare with cluster models.

Reviewer #2:

This paper proposes a trajectory inference method, Chronocell, incorporating RNA velocity and trajectory inference. The method aims to achieve the following few goals: 1. provide a unified model for RNA velocity and trajectory inference to improve intepretability of the model and results. 2. Separate between cell population that has a continuous trajectory structure from cell population that has discrete cluster-like structure. 3. Provide estimation of transcription related parameters such as degradation rates. I think the model proposed in the paper is interesting, though I have some concerns in the assumptions of the model. I also think the authors need more benchmarking results comparing with existing trajectory inference and RNA velocity methods to show benefits of the new approach.

Here are my specific comments:
1. The method assumes a known trajectory structure (a directed tree with known number of nodes, Page 25 line 525). I think this can be a big assumption but I'm not sure how much the authors need to assume from reading the methodology section. For example, does the method require knowing the shape of the directed tree (the number of branches at each stage) or just the number of nodes? Typically, even if there are biological prior knowledge, you would not know the exact shape of the trajectory structure, especially for complicated developmental processes. Most of the trajectory inference methods do not need to assume that. I think the authors need to clarify this assumption.
Additionally, many trajectory structures are not directed trees. For instance, it is common that there are diconnected cell states/types from the main branch (Figure 1c, Sealens et. al. Nature Biotech 2019). In Sec 2.5, the authors have applied Chronocell on a cell population with a cell cycle structure which is not a directed tree. I'm confused on how Choronocell can be applied to that scenario.

Thank you for pointing out the issue regarding the directed tree. In Choronocell, the trajectory structure was provided as a 2D array, with each lineage (path) represented as a row. Therefore, it does not have to be a directed tree. We have updated Sections 2.1 and 4.1.1 to clarify that we assume the topologies of the trajectory can be represented by a directed graph, with each lineage corresponding to a path within the graph. Consequently, cell cycle and disconnected structures are included.

We do require knowing the paths (and therefore the shape of the directed graph) for each fitting. However, different trajectory structures can be fitted to find the optimal one using model selection methods like AIC. This is similar to clustering, where the number of clusters is needed to fit each Gaussian mixture model, but model selection determines the optimal number of clusters. We have revised Section 2.2 to emphasize the demonstration of identifying the true structure when given an incorrect one in Figure 2, and comparing different structures to select the true disconnected structure via AIC/BIC in Figure S5.

The reviewer's question also touches upon a more general and far more challenging theme: statistical inference is heavily constrained by the set of models tested. We anticipate that, in the future, it may be possible to get a reasonable descriptive trajectory *shape* using simpler, computationally inexpensive methods, then use this shape to inform a Chronocell fit, then analyze its parameters and likelihoods to characterize whether the data are consistent with such a shape under the best-case scenario. This question is intimately related to the distinction between, e.g., K-means-like algorithms for clustering (which assume a particular structure) and adaptive algorithms (which robustly infer that structure). At this time, Chronocell is closer to the former extreme, but we have already made some inroads toward a more adaptive perspective by demonstrating the model selection extensions.

2. A question related to the above one. The authors claim that they can separate between cell population that has a continuous trajectory structure from cell population that has discrete cluster-like structure. How about cell population that has a subset of cells coming from a continuous trajectory and a subset of cells that come from discrete clusters? This scenario would happen often in practice.

This is a good point. We can test whether a continuous trajectory model or a discrete cluster model better fits the cell population using classic model selection methods such as AIC and BIC. Similarly, we can test whether a trajectory structure specifying part of it being a cluster fits the data better. We added a new figure (Figure S5) to demonstrate the inference for this scenario. We generated simulations containing a disconnected cluster and a bifurcation and tested the fitting. We compare it to a cluster model and a fully connected structure and AIC and BIC can correctly identify the true structures.

3. The authors assume constant transcription rate for each gene within a lineage and different transcription rate for each lineage. Are there any biological or empirical support on that assumption? Earlier empirical results seems to have shown that assumptions on the transcription rate can greatly affect the performance of RNA velocity method, can the authors provide further analysis on whether Chronocell is sensitive to the assumption on transcription rate?

We used a constant transcription rate for each state as a simplification, partially motivated by the observation that synthesis rates measured through metabolic labeling across the cell cycle may be roughly divided into a few stages, within which the rates appear to be at similar levels (Figure 2B, Battich et al., Science 2020). Indeed this is an oversimplification of transcription rates. To assess the impact of this assumption, we generate simulations in which transcription rates follow piecewise-exponential functions, allowing them to mimic linear or piecewise-constant behaviors with varying rate constants. We selected various rate constants to encompass the range from linear to piecewise-constant behaviors. Our results show that the accuracy remains satisfactory when the rate constants are comparable to or larger than the mRNA half-life. We have added a discussion on the sensitivity to this assumption in Section 2.3 as well as a new Figure S11.

The authors have also assumed that all selected genes have completely synchronized switch time of transcription rate. Can the authors provide some justification on this?

We assume completely synchronized switch time of transcription rates for simplicity in the synchronized model. As this assumption is likely not true in reality, we also have a desynchronized model, where each gene has its own switching time. The desynchronized model can be accurately fitted; however, it is more challenging to fit from scratch, so we use a warm start based on the results of the synchronized model (Figure S3). We have revised the text in Section 2.1 to clarify these points.

Conceptually, this idea is the trajectory generalization of discrete cell types (such that each cell belonging to a cell type has an identical average gene expression), or the deterministic-switching variant of certain trajectory models that describe cells transitioning between discrete states (each of which may have a distinct set of transcriptional parameters for each gene, as in http://dx.doi.org/10.1098/rsif.2020.0360).

4. As the authors discussed, some datasets have cells that are collected at a sequence of ordered time points. Can the method incorporate such information into the analysis?

Yes, Chronocell can incorporate time points information into the analysis by providing an informative sampling distribution prior. However, we do not have an answer for the key question of how to determine the appropriate form of this sampling distribution. For example, in a neuron dataset with cells sampled at five time points after stimulation, the choice of sampling distribution reflects our understanding of the process and cell heterogeneity at each time point. If we assume the cells respond homogeneously to the stimulation, we could use a delta distribution. However, if we account for heterogeneity within a time point, we may assume other distributions, such as exponential or normal. Furthermore, as shown in Figure S21, different prior assumptions can lead to varying outcomes. Therefore, while it is possible to incorporate time-point information, we do not have a definitive solution for how to do so. Conceptually, this is related to a variety of unsolved questions regarding the precise quantitative meaning of observing “earlier” and “later” cells in a dataset and the degree of timescale separation between this internal evolution and the externally imposed process.

5. Since there are many trajectory inference and RNA velocity methods and highly cited benchmarking paper on trajectory inference, I think the authors need to perform more benchmarking analysis to show the benefits of their method. For example, in the simulations, the authors can also generate synthetic data from existing software such as dyngen. In the real data analysis, the authors may compare with widely used methods such as slingshot and Monocle, existing methods combing trajectory inference and RNA velocity, and consider datasets that have more complicated trajectory structures. Currently, I have not seen a very clear evidence on why the users should use Choronocell instead of existing tools.

The advantage of Choronocell is that it provides biophysical interpretation through a biophysically motivated model of the dynamical processes, and a clear and rigorous inference framework. For benchmarking analysis, we have compared Chronocell with Slingshot, Monocle 3, diffusion pseudotime (scanpy) and recently published veloVI which also integrates trajectory inference with RNA velocity on simulated data in Figure 2, as well as simulations generated by dyngen. The results are presented in Figure S22 and S23 and mentioned in the discussion section. We found that other methods can not correctly recover true time on data generated under the Chronocell model (which is formally very close to a model proposed in the original RNA velocity publication) and have comparable or lower accuracy on dyngen simulation. We have also tested Chronocell on simulation with a more complex trajectory structure (Figure S10). However, for real data, benchmarking is more challenging due to the lack of ground truth. The closest approximation to ground truth is the experimental time in time series data, with short

intervals and a clear start point; however, such datasets are limited. Therefore, we test other methods on the neuron data, despite its expected structure not being complicated. The results are shown in Figure S24, which indicates that other methods either produce a distorted timeline or fail to capture the expected trend of inferred cell time progressing in sync with experimental time.

6. How does the method select DE genes along the estimated trajectory structure? Will the method avoid selecting false positive genes due to the fact that the process time (pseudotime) is estimated?

Since Chronocell provides transcription rate estimations, we directly select differentially expressed (DE) genes based on fold changes in transcription rates across states. To clarify, before selecting DE genes, we first filter genes based on goodness of fit (Section 4.3.2). We have also made slight modifications to the first paragraph of Section 2.4 for clarity.

Unfortunately, we do not have a way to calculate p-values rigorously for DE genes. There are two kinds of false positives for trajectory inference. The first one is demonstrated by identifying trajectory on cluster data, which we discussed in Figure S1 and S4 (Figure 2 previously). The second one happens when there is a trajectory but some genes do not change over time. The rigorous calculation of p-values for the second case is challenging because we are inferring the trajectory model together with parameters, conceptually similar to calculating p-values for features in principal components analysis (Taylor and Tibshirani, 2015, Statistical learning and selective inference), but with a more complicated model than PCA. By adopting a model-based approach, we do not need to go through the circular process of fitting trajectories and differential expression to interpret the result, but false positives caused by circularity still arise if we perform DE test naively based on the inferred time. We have added text in Introduction to clarify these. We anticipate the more challenging circularity problem will require considerable further improvements in sequencing statistics writ large. This problem has been recognized in previous studies in the context of clustering, as in, e.g., https://doi.org/10.1101/2023.07.21.550107. However, wider adoption, benchmarking, and evolution of proposed solutions is an outstanding problem, and we believe advances here will allow for more rigorous statistical treatment in the context of continuous trajectories.

7. A minor technical question: the notation of the model in section 4.1.2 is confusing. j stands for a gene, so \lambda_c should have an index of j instead of c? The method seems to assume that each gene as a gene specific transcription rate, degradation rate a splicing rate?

Apologies for the confusion, and thank you for catching this. Yes, we assumed that each gene has a gene-specific transcription rate, degradation rate, and splicing rate. We were initially using \lambda_c to denote the ODE solution for species c (unspliced/spliced). We have now updated Section 4.1.2 so that \lambda_c is written as \lambda^j_c where j is the gene index and c is the species index, and the notation for the capture rate q has been simplified as well.

8. Personally, I feel that some of the figures are too technical and might be hard to follow even if one has read the text multiple times. For example, I'm not sure if I understand the Chronocell part in Figure 2. Are the figures just illustrations? I can not understand the meaning of the numbers in each figure and there are no explanations. In figure 4, do the authors use Figure 4b to pick the best initialization? which one did they pick? Why does Figure 4 show that method performs very well and people should use it?

Thank you for the feedback; we have worked to improve the clarity of Figure 2. It was initially designed to illustrate that “pseudotime” can 'discover' superficially plausible dynamics on false positive cluster data, whereas with the Chronocell model, the cluster structure can be identified. This is essentially a negative control. The plots are inference results on simulated cluster data. To better explain and clarify the experiment and result. We have split Figure 2 into Figures S1 and S4, and have added more descriptions to the figures. Figure S1 demonstrates that descriptive methods cannot address false positives in a principled manner, whereas Figure S4 demonstrates that with a probabilistic model we can use classical model selection methods to compare with cluster models.

Figure 3b (Figure 4b previously) was meant to show that the Forebrain dataset is not suitable for Chronocell: the opposite direction has similar AIC values, which we suspect is due to the limited dynamical information, which is also supported by the results from the warm start in Figure 3c. We have revised Figure 3 and Section 2.4 to clarify it.
